# An efficient automated approach for accumulated dose estimation in prostate cancer radiotherapy

**DOI:** 10.1016/j.phro.2026.100942

**Published:** 2026-03-06

**Authors:** Maximilian Grohmann, David Krug, Andrea Baehr, Cordula Petersen, Manuel Todorovic, Sebastian Schäfer, Lukas Clemens Böckelmann, Elisabetta Gargioni

**Affiliations:** a Department of Radiotherapy and Radiation Oncology, University Medical Center Hamburg-Eppendorf, Hamburg, Germany; b Department of Radiation Oncology, University of Leipzig Medical Center, Leipzig, Germany; c Department of Radiation Oncology, University Medical Center Schleswig-Holstein, Kiel, Germany

**Keywords:** Prostate cancer radiotherapy, Dose accumulation, Offline adaptive radiotherapy, Hypofractionation, Cone-beam CT (CBCT), AI-based auto-contouring

## Abstract

**Background and Purpose:**

Anatomical variations during prostate radiotherapy can cause discrepancies between planned and delivered doses, which may become more pronounced in (ultra-)hypofractionated regimens. However, a standardized method for accumulated dose assessment is lacking. This study presented and validated an efficient automated workflow for retrospective dose accumulation in moderately hypofractionated prostate treatments, avoiding deformable image registration and daily dose recalculation.

**Materials and Methods:**

Twenty prostate cancer patients were analyzed retrospectively. The workflow combined daily cone-beam computed tomography (CBCT) imaging, online rigid registration, and artificial intelligence-based auto-contouring with planned dose data to estimate accumulated doses. Dose–volume metrics were analyzed for prostate, rectum, and bladder. Anatomical variations were assessed through volume and position analysis. Method accuracy was validated against CBCT-based dose recalculations in selected cases representing minimal and maximal variations.

**Results:**

The workflow required about one minute per fraction. Interfractional volume variability (coefficient of variation) was 35.2 %, 20.6 %, and 9.5 % for bladder, rectum, and prostate, respectively. Mean prostate displacement was 3.49 ± 2.07 mm, and the dice similarity coefficient averaged 0.83 ± 0.08. Bladder dose metrics (V_20Gy_, V_50Gy_, mean) differed by > 2 % in 45–49 % of sessions, rectal metrics in 35–39 %. Accumulated dose estimates agreed within ± 2 % of CBCT-based recalculations.

**Conclusions:**

Our automated workflow provides a robust, time-efficient method for monitoring interfractional dose variations in prostate radiotherapy. While constrained by CBCT image quality, the accuracy and practicality of the proposed method support its integration into clinical workflows, potentially improving offline adaptive decision-making.

## Highlights

• Automated one-minute workflow for accumulated dose estimation per fraction.
• Interfractional coefficient of variation: bladder 35 %, rectum 21 %, prostate 10 %
• Accumulated dose estimates agreed within ± 2 % of cone-beam based recalculations.
• Enabled efficient offline adaptive dose monitoring on standard hardware.

## Graphical abstract

**Figure:**
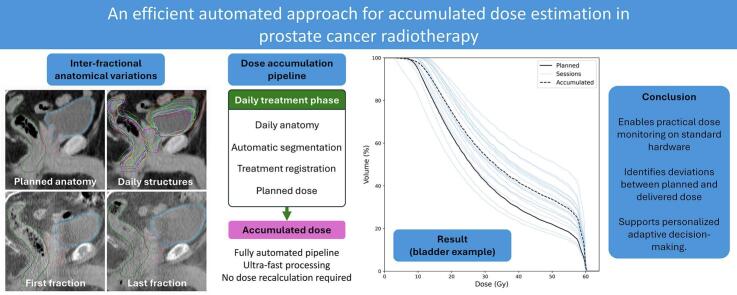

## 1 Introduction

Radiotherapy is a standard treatment for localized prostate cancer, aiming to deliver curative doses to the tumor while sparing adjacent organs of interest (OOI), such as bladder and rectum [1], [2], [3], [4]. Conventionally, treatment planning is based on a single planning computed tomography (pCT), which represents a static anatomy and remains unchanged throughout therapy [5]. Pelvic anatomy, however, is highly dynamic, as varying filling levels of bladder and rectum can shift and deform the prostate [6], [7], [8], [9], [10]. As a result, the delivered dose may deviate from the planned distribution, potentially affecting tumor control and side effects [6], [7]. Accumulated dose-volume histograms (DVHs) have been shown to correlate more closely with clinical outcomes than baseline DVHs [11].

This challenge is particularly relevant in hypofractionated treatment regimes, where higher doses per fraction magnify the impact of anatomical variations [12], [13], [14], [15], [16], [17]. Patient preparation protocols and daily image guidance help mitigate uncertainties, but variability cannot be eliminated [18], [19], [20], [21]. Current clinical decision-making still relies largely on qualitative, non-dose-based assessments, which may not fully capture the impact on dose distribution caused by anatomical changes [22], [23], [24]. Online adaptive radiotherapy (oART) manages daily anatomical variations by re-optimizing treatment plans for each fraction, but clinical uptake remained limited by resource-intensive workflows and specialized hardware. Moreover, adaptive decisions are often binary, leaving uncertainty regarding the cumulative impact of minor, unadjusted variations [25], [26], [27].

To estimate accumulated doses, two components are required: daily anatomical information and the corresponding dose distribution. However, manually replicating the full planning process for every treatment session was too time-consuming for routine clinical use. Alternatives, such as deformable image registration (DIR) [27] or auto-contouring, offer efficiency gains but introduce uncertainties or require substantial computational resources [28], [29], [30], [31], [32], [33], [34].

Given these limitations, efficient approaches to dose accumulation that are both accurate and clinically feasible are needed. In this work, we presented an automated workflow that combines AI-based auto-contouring with baseline dose data to estimate fractional and accumulated organ doses without DIR or daily dose recalculation. We retrospectively analyzed hypofractionated prostate treatments at our institution with three objectives: (1) compare baseline versus accumulated doses for target and OOI, (2) quantify interfractional anatomical variations, and (3) validate the approach against cone-beam computed tomography (CBCT)-based dose recalculations.

## 2 Materials and Methods

This retrospective analysis used previously acquired, fully anonymized clinical data without intervention. According to institutional and national regulations, retrospective analyses based exclusively on anonymized data do not require ethics committee consultation or approval. All patients had provided written informed consent for scientific data use.

### 2.1 Study design and patient cohort

This retrospective study included 20 patients with localized prostate cancer treated with external-beam radiotherapy using the CHHiP protocol [12], i.e., with a total dose of 60 Gy, delivered in 20 fractions, to the prostate. Patients were instructed to maintain a comfortably full bladder and an empty rectum during CT simulation and throughout all radiotherapy treatment sessions. However, no standardized bladder-filling protocol (e.g., drinking a fixed amount of fluid before pCT and treatment) or rectum preparation (e.g., enema) was applied.

### 2.2 Treatment planning and imaging

All treatment plans were created using the Eclipse Treatment Planning System (v16.1, Varian Medical Systems, Palo Alto, CA, USA). Treatments were delivered on a Varian TrueBeam STx linear accelerator (linac) equipped with kV-CBCT imaging capability. Daily CBCT scans were acquired for patient positioning verification (see Supplementary Material A for acquisition and reconstruction parameters).

### 2.3 Workflow and dose accumulation method

**Fig. 1:**
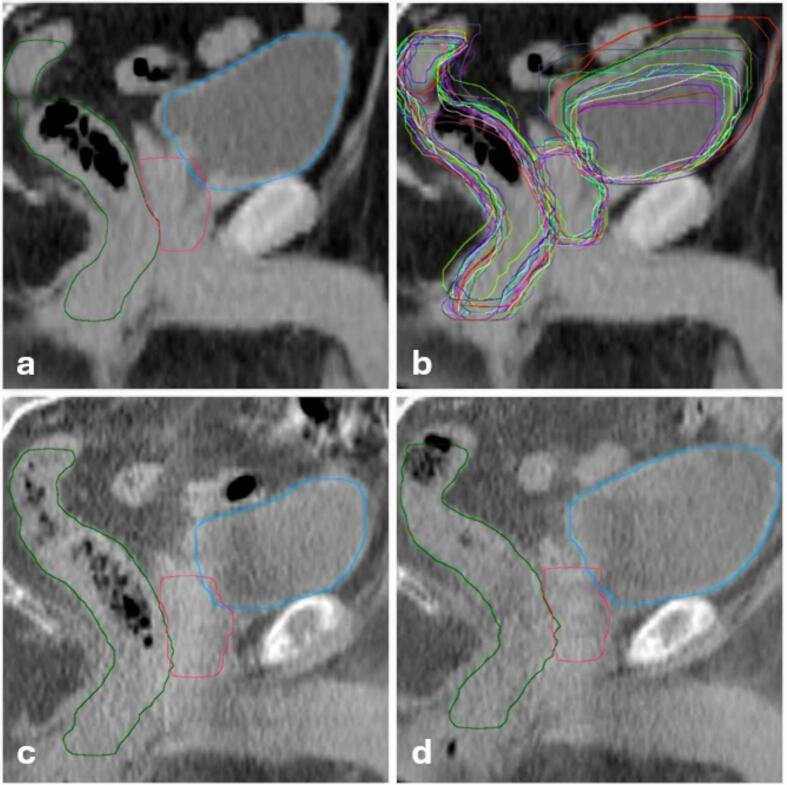
Sagittal views of the pelvis on the planning CT (a,b) and daily CBCTs (c,d), illustrating automatically contoured prostate (red), bladder (blue), and rectum (green) for patient 7. The top‐left panel shows the planning CT with its initial auto‐contours, while the top‐right panel overlays auto‐contours for each treatment day (random colors) on the same planning CT. The bottom‐left and bottom‐right panels display CBCT images from the first and last (20th) fraction, respectively, each with daily auto‐contours for these organs. (For interpretation of the references to colour in this figure legend, the reader is referred to the web version of this article.)

The developed workflow consisted of three sequential processing steps. Initially, daily CBCTs and their corresponding 4x4 online registration matrices, obtained during patient positioning, were exported using a custom C# script developed utilizing the Eclipse Scripting API (v16.1). Subsequently, Limbus Contour AI (v1.8.0; Limbus AI Inc, Regina, SK, Canada) was employed to automatically generate contours for body, bladder, rectum, and prostate on both pCT and daily CBCTs (Fig. 1). All artificial intelligence (AI)-generated contours were visually screened for plausibility; no exclusions were required.

**Fig. 2:**
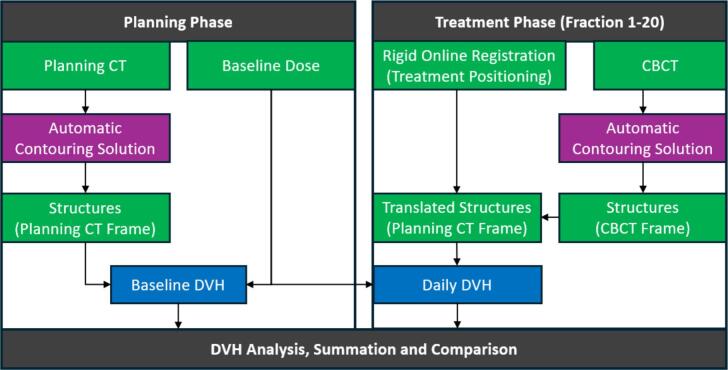
Overview of dose estimation pipeline. The workflow is divided into planning phase (left) and treatment phase (right). Structures are automatically contoured on both Planning CT and daily CBCTs (purple), then aligned using online registration matrices. Green boxes represent DICOM data in their respective coordinate frames, while blue boxes show the resulting DVH calculations used for dose analysis. (For interpretation of the references to colour in this figure legend, the reader is referred to the web version of this article.)

The final step utilized a custom Python-based pipeline (v3.11.7) that integrated pydicom [35] and dicompyler-core [36] libraries for DICOM data processing (Fig. 2). This pipeline extracted axial contour points from the CBCT structures and applied the online registration matrices to perform rigid translation into the pCT coordinate system (see Supplementary Material B for workflow details and open-source implementation). By combining these transformed structures with the dose distribution of the original treatment plan, we calculated daily DVHs, which then accounted for anatomical variations and setup corrections. For comprehensive analysis, we automatically generated an additional structure with a uniform 1-cm margin around the prostate to approximate a planning target volume (PTV). All processing steps were performed on a standard desktop computer (Intel Core i7 processor, 16 GB RAM) without requiring dedicated GPU acceleration.

### 2.4 Geometric and dose–volume analysis

Analyses were tailored to the anatomical characteristics and clinical relevance of each structure, with positional and shape-based metrics applied to the prostate, and volume- and dose-based metrics applied to bladder and rectum.

To assess the impact of anatomical variations on treatment delivery, we analyzed volumetric changes in the prostate, bladder, and rectum over all fractions, including relative volume deviations compared to the pCT. Additionally, we quantified the center of mass distance (CMD) of the prostate to evaluate positional shifts. To assess both position and shape variations of the prostate, we calculated the dice similarity coefficient (DSC) between daily contours and planning CT contours. The DSC was defined as shown in Equation (1) [37], [38]:

(1) DSC=2|A∩B|/(|A|+|B|)

where A and B represent the volumes of the daily and pCT contours, respectively. This metric ranges from 0 to 1, with 1 indicating perfect overlap and 0 indicating no overlap, providing a comprehensive measure of geometric similarity that accounts for both positional and shape differences.

Dose–volume analysis was performed following the ICRU Report 83 dose reporting recommendations [39], including D_98 %_, D_50 %_, D_2 %_, mean dose, and the relative volumes receiving at least 20 Gy and 50 Gy (V_20Gy_, V_50Gy_). Throughout this manuscript, D_x%_ denotes the minimum dose delivered to x % of the structure volume, while V_xGy_ describes the percentage of volume receiving at least x Gy; unit brackets are omitted in the text for readability.

The planned dose distribution (calculated on the pCT) was used as the baseline. We compared session and accumulated dose-volume metrics against baseline values, including D_98 %_ (near-minimum dose), D_50 %_ (median dose), D_2 %_ (near-maximum dose), V_20Gy_, V_50Gy_, and mean dose. Relative differences between session and baseline metrics were calculated as percentage changes normalized to the planned values. Furthermore, we generated dose-volume histograms (DVHs) for all structures to illustrate both individual fraction variations and accumulated dose distributions across the treatment course. Deviations of the dose-volume metrics between treatment plan and daily session were considered relevant if they exceeded a threshold of 2 %, pragmatically chosen to exclude minor fluctuations from the analysis.

### 2.5 Statistical analysis

To investigate dose variations caused by anatomical changes, we performed a Pearson correlation analysis between daily organ volume changes (relative to pCT) and corresponding mean dose differences for bladder and rectum. This analysis was performed across all fractions and patients. The statistical analysis was conducted in Python using SciPy [40], with correlation strength interpreted according to Cohen’s conventions (|r| <0.3: weak; 0.3–––0.5: moderate; > 0.5: strong) [41].

Due to skewed distributions and outliers in volume-related and relative dose parameters, these variables are reported as median and interquartile range (IQR). Approximately symmetric variables are presented as mean ± standard deviation (SD).

### 2.6 Validation using CBCT dose recalculation

To validate the proposed method, we selected three patients from our cohort based on the coefficient of variation (CV) of their body contour volumes, calculated as shown in Equation (2):

(2) $$\mathrm{CV}=\frac{\sigma }{\mu }\times 100\%$$

where σ is the SD and μ the mean of the body volume over all fractions. We considered the body contour for selecting validation cases rather than prostate, bladder, or rectum, since these organs exhibit similar tissue densities, making volume changes less impactful for dose estimation. In contrast, variations in the overall body contour could influence the dose calculation due to differences in beam attenuation and scattering. For the selected cases, daily CBCT-based dose recalculations were performed using our clinical workflow, where the original treatment plan was copied onto the CBCT dataset, and dose was recalculated using the Hounsfield Unit (HU)-density conversion curve of the CBCT [42]. We then assessed the differences between our approach and the CBCT-based recalculations for key dose-volume parameters.

## 3 Results

The implemented workflow achieved efficient processing times − approximately one minute per fraction (10 s for data export, 40 s for AI-based contouring, and 10 s for analysis). For a complete treatment course of 20 fractions, this resulted in approximately 20 min of processing time per patient.

**Fig. 3:**
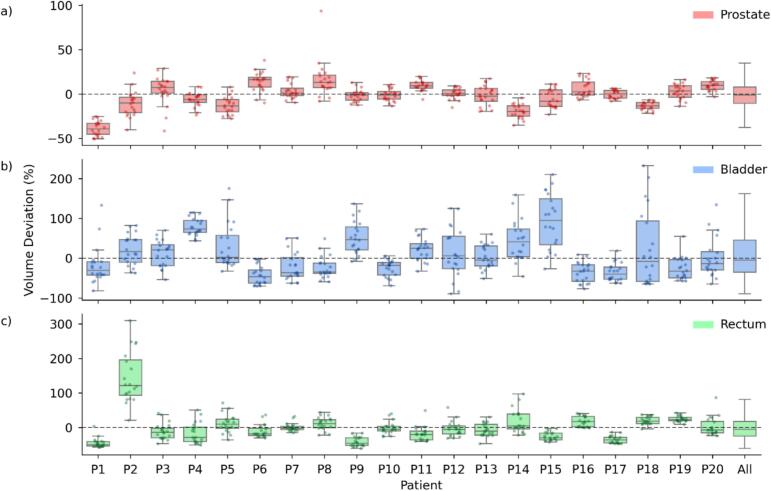
Relative volume deviations of prostate, bladder, and rectum across treatment sessions. Box-and-whisker plots illustrating the relative volume deviations of the prostate (a, red), bladder (b, blue), and rectum (c, green) across treatment sessions compared to the planning CT for each patient (P1–P20). The rightmost 'All' column summarizes the overall distribution for each organ. The boxes represent the interquartile range (IQR, 25th to 75th percentile) with the median as a central line, while whiskers extend to the most extreme data points within 1.5 × IQR. (For interpretation of the references to colour in this figure legend, the reader is referred to the web version of this article.)

The average pCT volumes were 42.2 ± 14.6 cm^3^ for prostate, 205.9 ± 131.8 cm^3^ for bladder, and 73.5 ± 23.2 cm^3^ for rectum. Interfractional volume variability differed substantially between organs, with the highest CV observed for the bladder (35.2 %), followed by the rectum (20.6 %), while the prostate remained comparatively stable (9.5 %). Despite these summary measures, individual fractions showed pronounced deviations from baseline, with bladder volumes ranging from + 232 % to − 89 % relative to pCT. The rectum showed an overall tendency toward volume reduction compared to baseline (Supplementary Material C). Across all patients, the median (IQR) volume changes were − 1.0 % (−10.5 to 8.0 %) for the prostate, −4.6 % (−35.1 to 45.4 %) for the bladder, and − 5.0 % (−25.4 to 17.7 %) for the rectum relative to the respective planning volumes (see Fig. 3, column 'All').

**Fig. 4:**
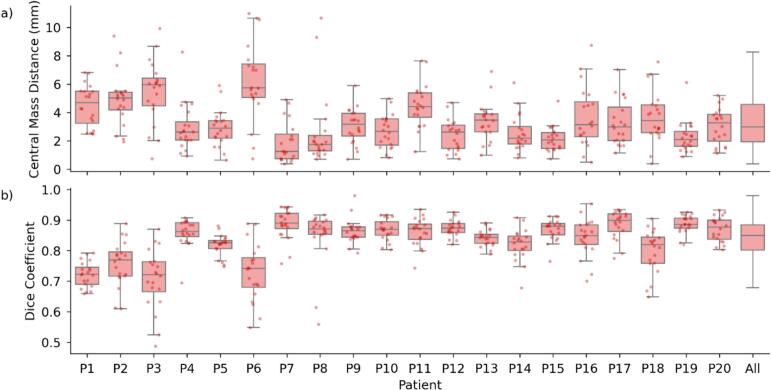
Prostate position and shape variation across treatment sessions. (a) Box-and-whisker plots showing the center of mass distance (CMD) between treatment sessions and the planning CT for the prostate across all patients (P1-P20). (b) Corresponding dice similarity coefficients (DSC, Equation (1), a measure for the volumetric overlap between daily contours and planning CT contours. For both plots, boxes represent the interquartile range (IQR, 25th to 75th percentile) with the median as a central line, while whiskers extend to the most extreme data points within 1.5 × IQR. Individual session measurements are shown as scattered points, jittered horizontally for better visibility. The rightmost 'All' column summarizes the metrics across all patients. CMD values reflect the three-dimensional displacement of the prostate's center of mass over the treatment course, while dice similarity coefficients quantify the geometric similarity between daily and planning contours.

The daily prostate position varied relative to the pCT, with a mean CMD of 3.35 ± 1.93 mm (Fig. 4); maximum displacements during single sessions reaching about 10 mm. For patients P3 and P6, the median CMD was the largest (nearly 6 mm). Moreover, the DSC between daily and pCT contours yielded a mean value of 0.83 ± 0.08. Also in this case, while for most patients this value was close to 0.9, we observed average DSC smaller than 0.8 for patients P1, P3, and P6.

**Fig. 5:**
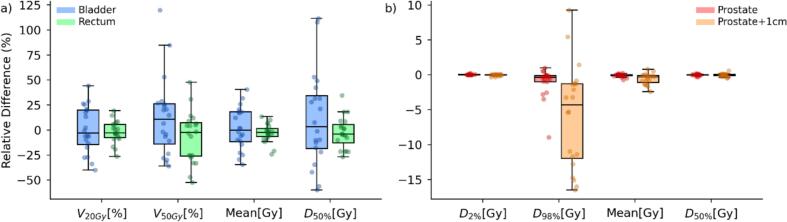
Percentage differences between accumulated and planned dose–volume parameters. Box-and-whisker plots illustrate the relative differences between accumulated and planned dose metrics for organs of interest (a) and target volumes (b) across all treatment fractions. Boxes represent the interquartile range (25th–75th percentile), with the median shown as a central line, while whiskers extend to the most extreme data points within 1.5 × IQR. Individual points indicate session-specific values. Positive differences reflect higher accumulated doses compared to the plan, while negative values indicate underdosage.

Accumulated dose-volume metrics deviated substantially from planned values, particularly for the bladder and rectum. For OOI (Fig. 5a), the bladder exhibited the largest deviations, particularly in V_20Gy_ and V_50Gy_, with relative changes exceeding 50 % in some cases. Bladder metrics exceeded the 2 % threshold in 45 % of cases for V_20Gy_, 49 % for V_50Gy_, and 45 % for mean dose, respectively. The rectum showed moderate deviations, with V50Gy and mean dose remaining closer to the planned values, though still exceeding the threshold in 39 % of cases for V_20Gy_ and V_50Gy_, and 35 % for mean dose. For the target volume (prostate + 1 cm margin), the near-minimum dose (D_98 %_) showed the largest deviation, reaching up to −15 %, while mean and near-maximum doses (D_2 %_) remained within a narrow range of the planned values (Fig. 5b).

**Fig. 6:**
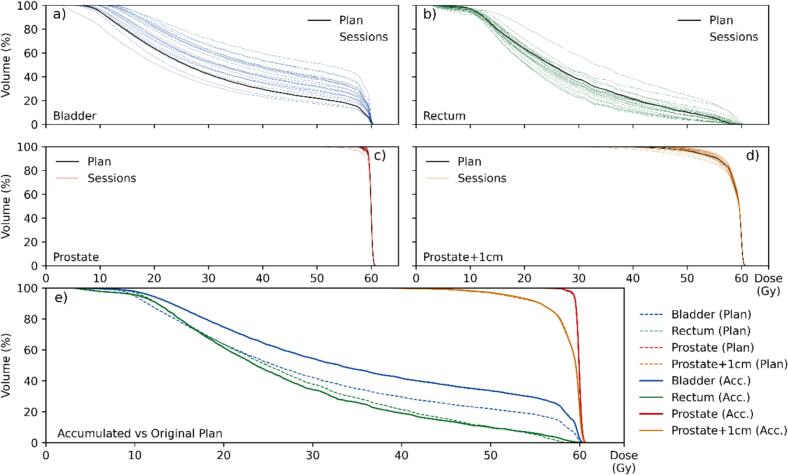
Accumulated DVH assessment for Patient 17. (a–d) Daily DVHs for bladder, rectum, prostate, and prostate + 1 cm. Black lines represent the planned dose distribution, while colored lines indicate DVHs from individual treatment sessions. (e) Comparison of the estimated accumulated DVHs (solid lines) with the original treatment plan (dashed lines) for all structures.

For the representative patient P17, mean volume deviations relative to baseline were −35.9 % (bladder), −35.1 % (rectum), and −0.1 % (prostate) (Fig. 6). The accumulated dose for the target volumes (prostate and prostate + 1 cm) matched closely with the baseline. The bladder received a higher accumulated dose than planned, while the rectum dose remained similar to the planned one, despite showing larger variations between sessions. Individual analyses for all 20 patients were provided in Supplementary Material D.

Inverse correlations were observed between relative organ volume changes and corresponding mean dose differences. According to Cohen’s conventions, the bladder exhibited a strong negative correlation (r = -0.61), while the rectum showed a moderate negative correlation (r = -0.31).

Comparison with CBCT-based dose recalculations for three representative patients demonstrated close agreement with the accumulated dose estimates. Mean differences in dose metrics remained within ± 1.5 % for most parameters across all evaluated fractions. The largest deviations were observed for Patient P2, particularly for the rectum (D_50 %_: −2.5 %) and bladder (D_50 %_: −2.2 %). Differences for the prostate remained below 2 % even in the most challenging case. Across all evaluated patients and structures, mean differences remained within ± 1.1 % (see Supplementary Material E, Table S1).

## 4 Discussion

This study evaluated a fully automated workflow for dose accumulation in twenty prostate cancer patients. The proposed method, which utilized AI-based contouring and rigid online registration, achieved high processing efficiency and demonstrated strong agreement with CBCT-based dose recalculations. Key findings included substantial interfractional volume variations for the bladder and rectum, leading to relevant dose-volume metric deviations from treatment plan.

In our study, the mean prostate CMD was 3.5 ± 2.1 mm, moderately lower than the 4.6 ± 3.5 mm reported by Frank et al. [43]. This discrepancy may reflect setup protocols: their weekly CT-on-rails versus our daily CBCT. Image quality differences could further affect contouring and registration accuracy. Geometric agreement between pCT and daily CBCT contours, assessed via DSC, showed strong consistency with a mean of 0.83 (range: 0.49–––0.98), consistent with Thor et al. [44], who reported mean DSC of 0.80 (range: 0.67–––0.87) using DIR. Together, CMD and DSC characterize interfractional anatomical consistency relative to the baseline, while implicitly reflecting combined effects of setup uncertainties, anatomical changes, contouring performance, and registration accuracy. Despite these interacting factors, the observed values remained within clinically reasonable limits, supporting the feasibility of the proposed framework.

Regarding differences in mean organ doses, Huang et al. [6] reported deviations of 18 % (bladder), 22 % (rectum), and 2 % (prostate) relative to baseline, while our analysis yielded 25 %, 12 %, and 0 %, respectively. Fuchs et al. [7] observed normalized mean bladder doses ranging from 58 − 160 % and rectal doses from 87 − 118 %, closely matching our ranges of 65–––140 % and 75–––113 %. These findings also confirmed that, even under daily CBCT guidance, substantial deviations from planned doses occur, particularly for bladder and rectum.

Beyond these physical deviations, biological implications warrant attention. As shown in Fig. 6, accumulated rectal dose largely matched the plan, yet daily fluctuations were substantial. Scaife et al. [10] reported similar results, with daily EUD deviations of up to −17 % and + 10 % relative to baseline. Such transient overdoses or underdoses may contribute to side effects even when accumulated doses remain close to planned values, emphasizing the need to assess daily variations in addition to cumulative distributions.

Byrne et al. [45] showed in an oART study (366 fractions, 18 patients) that adapted plans were preferred in 95 % of fractions and achieved more treatment goals in 78 % of cases compared to non-adapted plans. Our findings are consistent with these results, as bladder and rectum metrics exceeded a 2 % deviation threshold in ≥ 35 % of fractions. Similarly, Bohoudi et al. [11] demonstrated that accumulated delivered dose outperformed baseline DVHs in predicting acute urinary adverse events in prostate SBRT (area under the curve, AUC: 0.71–––0.75 vs. 0.53–––0.62). These data support the feasibility and clinical relevance of systematic dose monitoring, facilitating identification of relevant deviations and informing adaptive replanning or retrospective dose evaluation.

The proposed workflow has several advantages. By avoiding DIR and daily dose recalculation, it enables minute-scale runtimes on standard hardware, supporting integration into clinical workflows. Because the workflow relies on standard DICOM data and routine CBCT imaging, it can be implemented at other institutions. It remained robust when CBCT scans are incomplete in the cranio-caudal direction or truncated laterally, provided relevant structures remain visible. Validation against full CBCT-based recalculations showed differences within 1.5 % for key parameters, even in cases with substantial anatomical variation.

Certain limitations must be acknowledged. Accuracy depended on CBCT quality, which influenced both manual and automated contours. Our pipeline accounted for pre-treatment anatomical volumes via CBCT contouring, but rigid registration remains a compromise, as daily anatomy cannot be identical to baseline. DIR approaches model deformation explicitly but introduce their own uncertainties, particularly related to image quality, registration accuracy, and algorithm-dependent variability. Furthermore, intrafraction motion and transient anatomical changes after setup were not captured, potentially requiring complementary strategies, such as in-vivo dosimetry [46]. Additionally, the correlation analysis between volume and dose changes was performed across all fractions. While this effectively illustrates global physical trends, it did not account for the statistical clustering of fractions within individual patients. For sites with marked density heterogeneity or rapid anatomical change (e.g., lung, head & neck), explicit dose recalculation or more advanced adaptive techniques may be needed.

With respect to contouring, while auto-contouring tools have demonstrated reliable performance on pCTs [47], CBCT contours require further validation against expert delineations. Nevertheless, within our framework, auto-contouring reduced interobserver bias and ensures consistency across fractions. Consistency was further reinforced by identical imaging presets and devices across all CBCTs. Previous work suggests that CBCT deep-learning contours can achieve quality comparable to human observers [48]. Given the magnitude of baseline vs. daily DVH differences in our data, small contouring uncertainties are likely less critical than the anatomical changes themselves.

Future improvements in CBCT − such as higher soft-tissue contrast and reduced artifacts from next-generation systems − may further enhance auto-contouring performance [49], [50]. Rapid acquisition times of ring accelerators can additionally help mitigate motion-related uncertainties, improving integration into adaptive workflows [51]. Looking forward, extending this pipeline to near-real-time adaptive use, evaluating additional disease sites, and leveraging baseline vs. accumulated DVH comparisons for NTCP modeling could further broaden its clinical utility.

The presented method enabled comprehensive assessment of dose accumulation in radiotherapy and could be implemented efficiently with minimal time and infrastructure requirements. The retrospective analysis for prostate cancer patients revealed substantial differences between baseline and accumulated doses − particularly for the bladder − accompanied by significant volume changes. By comprehensively capturing these variations, the workflow allows for quantified intervention thresholds, supports clinical decision-making, and may facilitate more personalized treatments. While online-adaptive techniques remain limited, this fully automated approach provides an additional, efficient option for offline adaptation and enables dose reporting based on the accumulated rather than the planned dose.

**Declaration of generative AI and AI-assisted technologies in the manuscript preparation process**

During the preparation of this work, the author used *ChatGPT (GPT-5, OpenAI)* to assist with English language proofreading and minor grammar improvements. The tool was used solely to enhance linguistic clarity; all scientific content, analyses, and interpretations were developed, verified, and validated by the author. After using this tool, the author thoroughly reviewed and edited the text and takes full responsibility for the content of the published article.

## CRediT authorship contribution statement

**Maximilian Grohmann:** Conceptualization, Data curation, Formal analysis, Investigation, Methodology, Project administration, Resources, Software, Supervision, Validation, Visualization, Writing – original draft, Writing – review & editing. **David Krug:** Conceptualization, Project administration, Resources, Supervision, Writing – review & editing. **Andrea Baehr:** Conceptualization, Writing – original draft, Writing – review & editing. **Cordula Petersen:** Conceptualization, Resources, Writing – review & editing. **Manuel Todorovic:** Resources, Writing – review & editing. **Sebastian Schäfer:** Conceptualization, Writing – original draft, Writing – review & editing. **Lukas Clemens Böckelmann:** . **Elisabetta Gargioni:** Conceptualization, Data curation, Methodology, Supervision, Validation, Writing – original draft, Writing – review & editing.

## Declaration of competing interest

The authors declare the following financial interests/personal relationships which may be considered as potential competing interests: David Krug has received honoraria from Astra Zeneca, best practice onkologie, ESO, ESMO, Gilead, med update, Merck Sharp & Dohme, Novartis, onkowissen, Pfizer and PINK gegen Brustkrebs, served on advisory boards for Gilead and has received institutional research funding from Stiftung Deutsche Krebshilfe and Merck KGaA.
