# Supplementary material for: An efficient automated approach for accumulated dose estimation in prostate cancer radiotherapy
Source: Phys Imaging Radiat Oncol. 2026 Mar 6;38:100942. doi: 10.1016/j.phro.2026.100942 (PMC12993423; doi:10.1016/j.phro.2026.100942)
Supplement: Supplementary Data 1 [file mmc1.pdf]

Supplementary Material for ‚An efficient automated approach for accumulated dose estimation in prostate cancer radiotherapy‘

- Supplementary Material A – CBCT protocol parameters
- Supplementary Material B – Script Pipeline for Structure Transformation and Data Management Overview (Open Source)
- Supplementary Material C – Absolute Volume Variations
- Supplementary Material D – Patient-specific DVH Analyses
- Supplementary Material E – Validation of Dose Accumulation Against CBCT Recalculations

Supplementary Material A – CBCT protocol parameters

Daily CBCT scans were acquired using a standardized pelvic kV-CBCT protocol on a Varian TrueBeam STx linear accelerator. Acquisition parameters were kept constant across all patients and fractions to ensure consistent image quality for patient positioning, rigid registration, and subsequent contouring analyses. Scans were performed with a tube voltage of 125 kV and a tube current–time product of 1080 mAs, using a full gantry rotation with 900 projections and a total acquisition time of 60 s. Images were reconstructed using the system’s default reconstruction method with a standard reconstruction filter, a matrix size of 512 × 512 pixels, and a slice thickness of 2.0 mm. These acquisition and reconstruction settings reflect routine clinical practice and were selected to provide a balanced trade-off between image noise and spatial resolution. As CBCT acquisition and reconstruction parameters can influence soft-tissue contrast, contour definition, and registration accuracy, maintaining fixed protocol settings was essential to minimize systematic variability across fractions.

Supplementary Material B – Script Pipeline for Structure Transformation and Data Management Overview

To ensure reproducibility and transparency, a simplified open-source version of the core functions used for structure transformation in this study is available at
https://github.com/Kiragroh/Transform_RTSTRUCTwithREG
This implementation includes the key routines from main_prepare_transformStructures.py, which perform rigid alignment and conversion of daily CBCT-derived structure sets into the planning CT coordinate system using the transformation matrix contained in the corresponding DICOM registration (REG) file. It can optionally copy the Frame of Reference (FoR) from a planning RTSTRUCT to harmonize metadata and ensure consistency across datasets.

1. Data Management and Preparation

Before transformation, new imaging data of watched patients are retrieved and prepared automatically. Relevant DICOM-RT datasets (Limbus Structure Set from the planning CT, baseline dose, and baseline plan) and daily CBCT images are exported and organized. The CBCT slices are then transferred to the AI-based auto-contouring solution, where new daily structure sets of the bladder, rectum, prostate, and body are created.

2. Structure Transformation

After auto-contouring, the transformation process is performed using the functions described in *Transform_RTSTRUCTwithREG*. These routines execute rigid transformation of all contours into the planning CT coordinate frame and generate datasets ready for accumulated dose computation and visualization.

3. DVH Computation and Comparison

Dose–volume histograms (DVHs) were computed directly from the transformed RTSTRUCTs and the baseline dose using the *dicompyler-core* library [37]. The resulting dose metrics were compared with baseline plan values and, in selected validation cases, with full CBCT-based dose recalculations. All DVH computations were performed using consistent logic to ensure reproducibility and comparability across patients and treatment fractions.

Bonus: Eclipse scripts for structure comparisons.

<u>Supplementary Material C – Absolute Volume Variations</u>

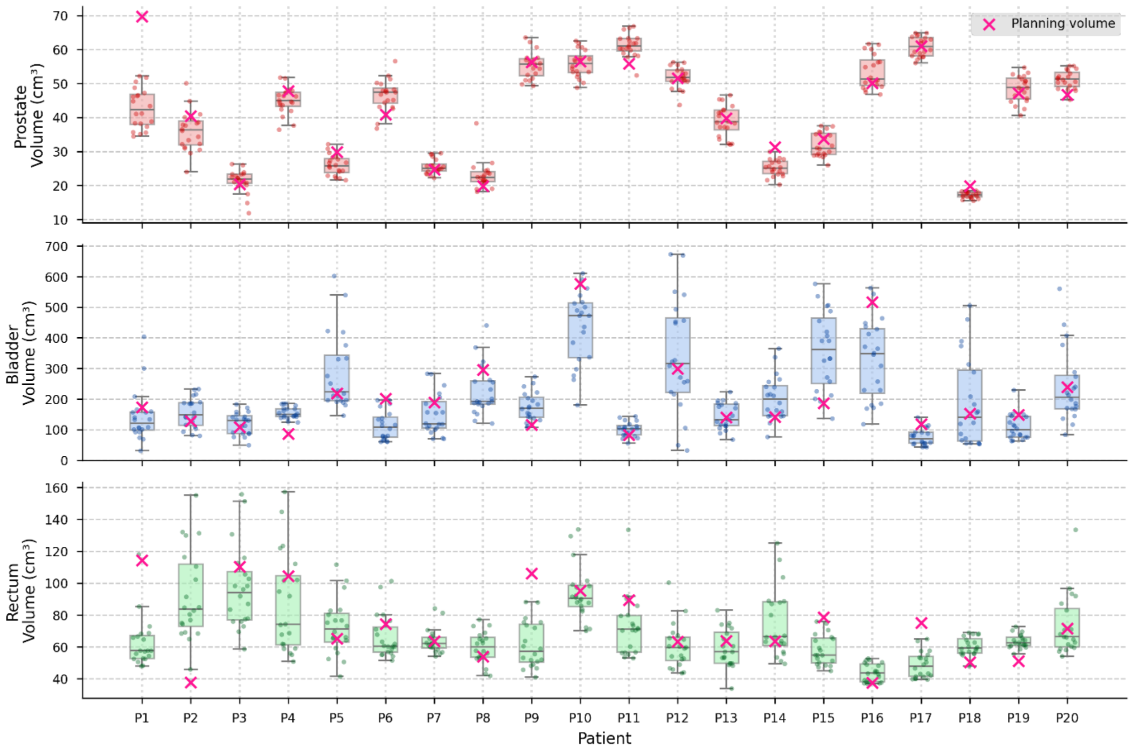

Figure S1: Volume variations of prostate, bladder, and rectum across treatment sessions. Box-and-whisker plots illustrating the variations in prostate (top, red), bladder (middle, blue), and

rectum (bottom, green) volumes across treatment sessions for each patient (P1–P20). The boxes represent the interquartile range (IQR, 25th to 75th percentile) with the median as a central line, while whiskers extend to the most extreme data points within 1.5×IQR. Individual session measurements are displayed as scattered points, jittered horizontally for visibility. The magenta crosses indicate the volume from the planning CT for each patient.

Supplementary Material B – Patient-specific DVH Analyses

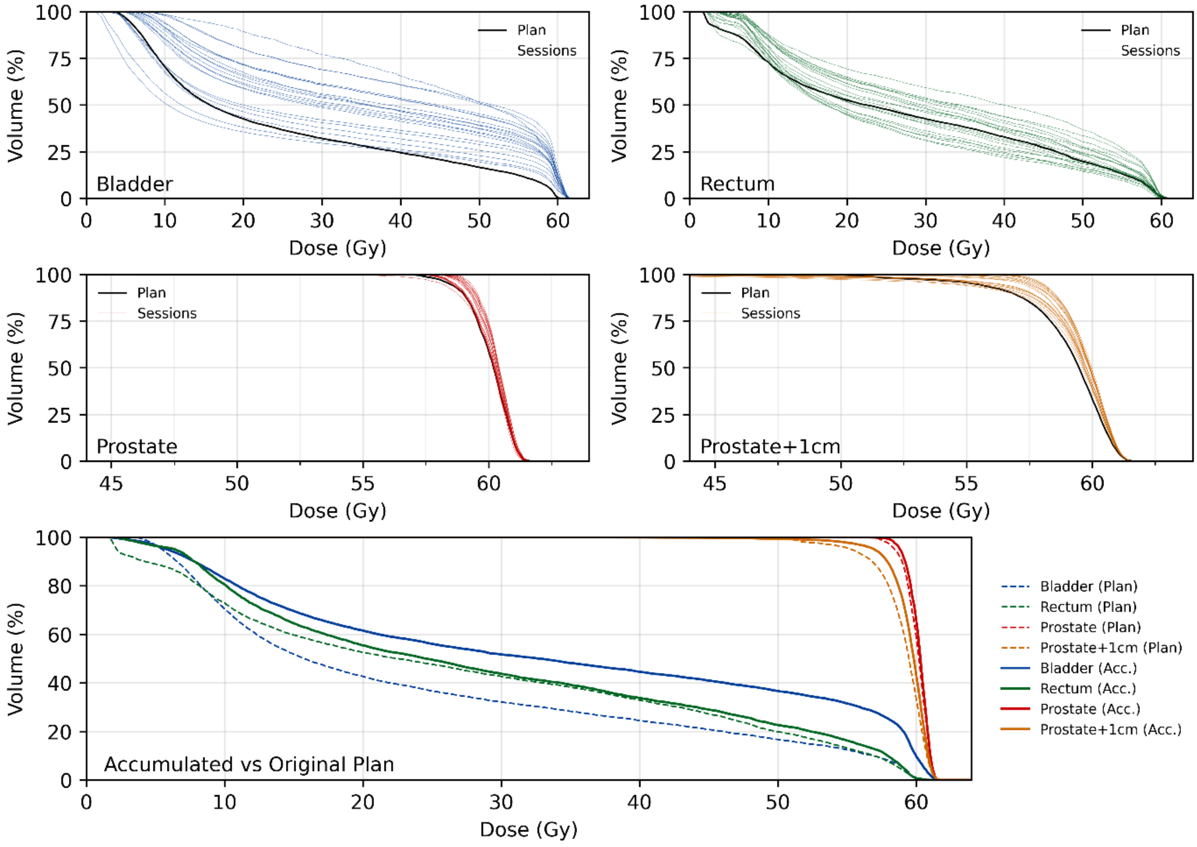

Figure S2. Accumulated DVH assessment for Patient P1.

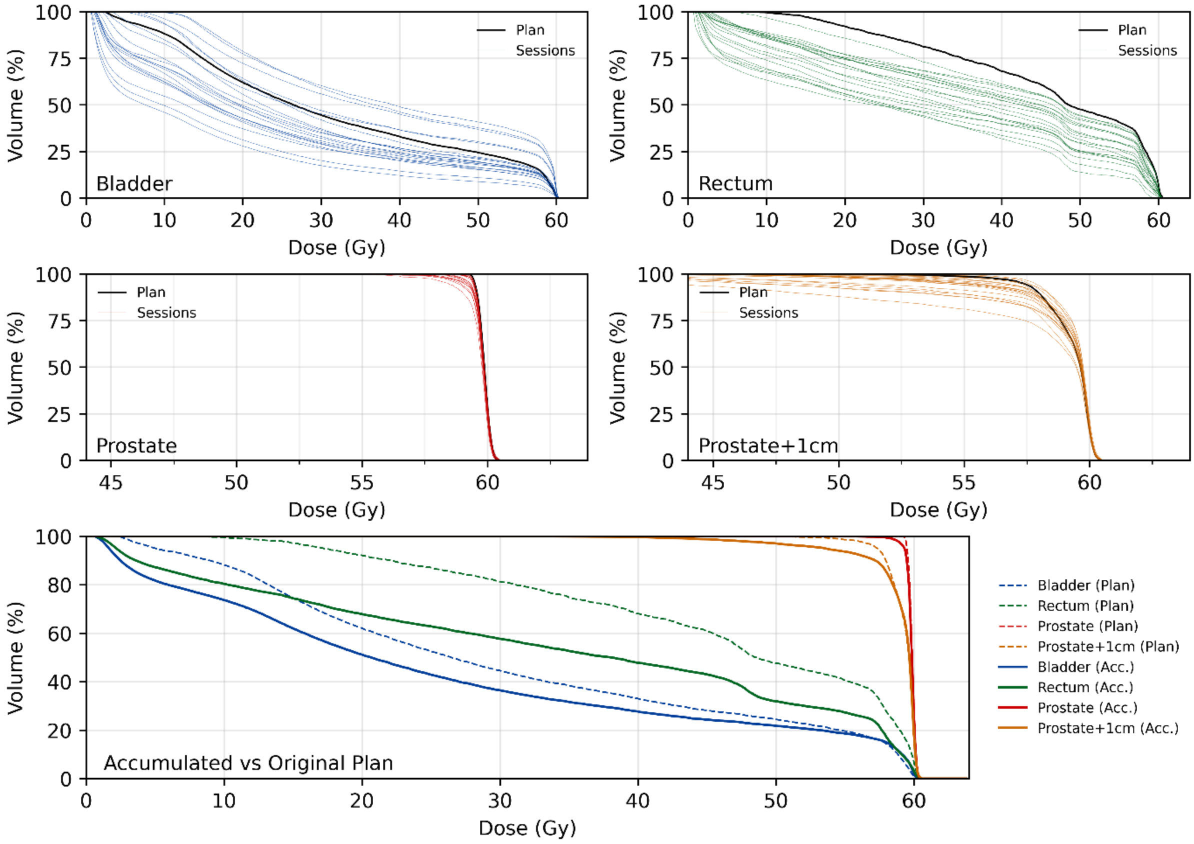

Figure S3. Accumulated DVH assessment for Patient P2.

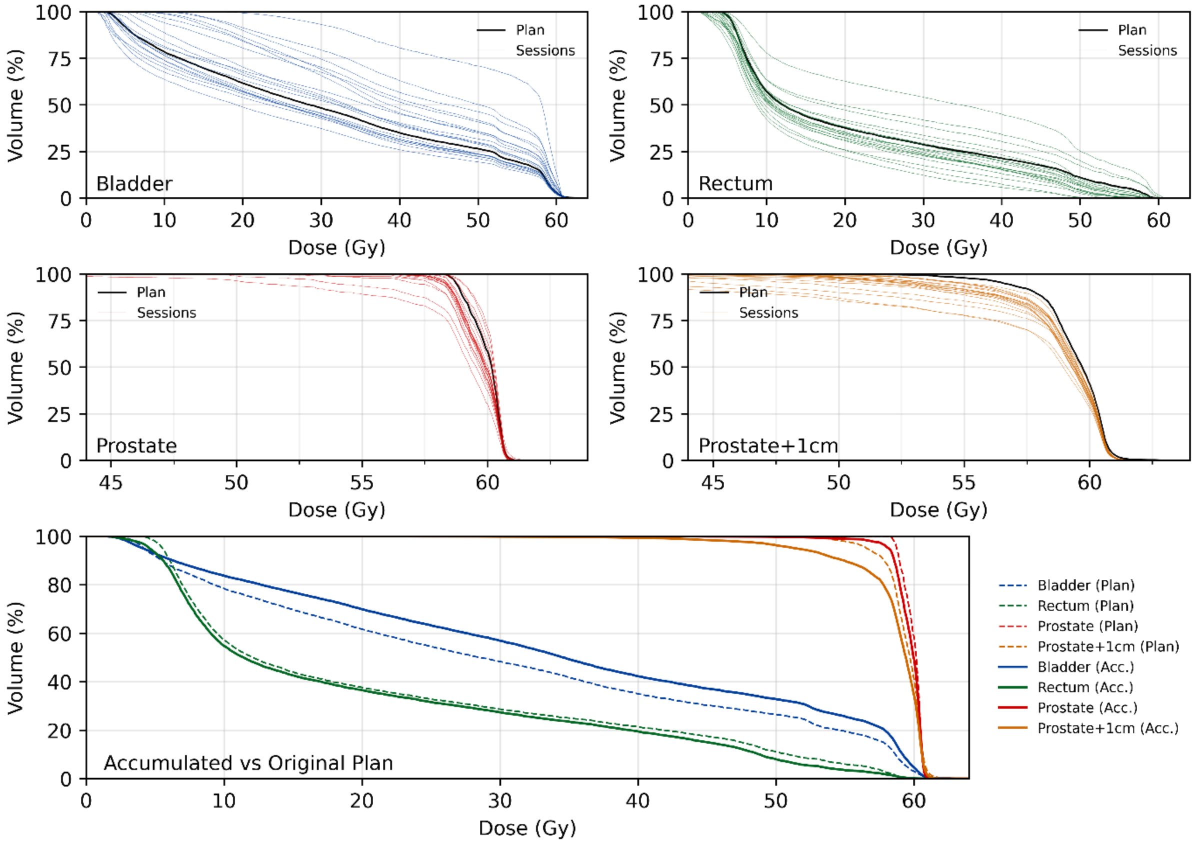

Figure S4. Accumulated DVH assessment for Patient P3.

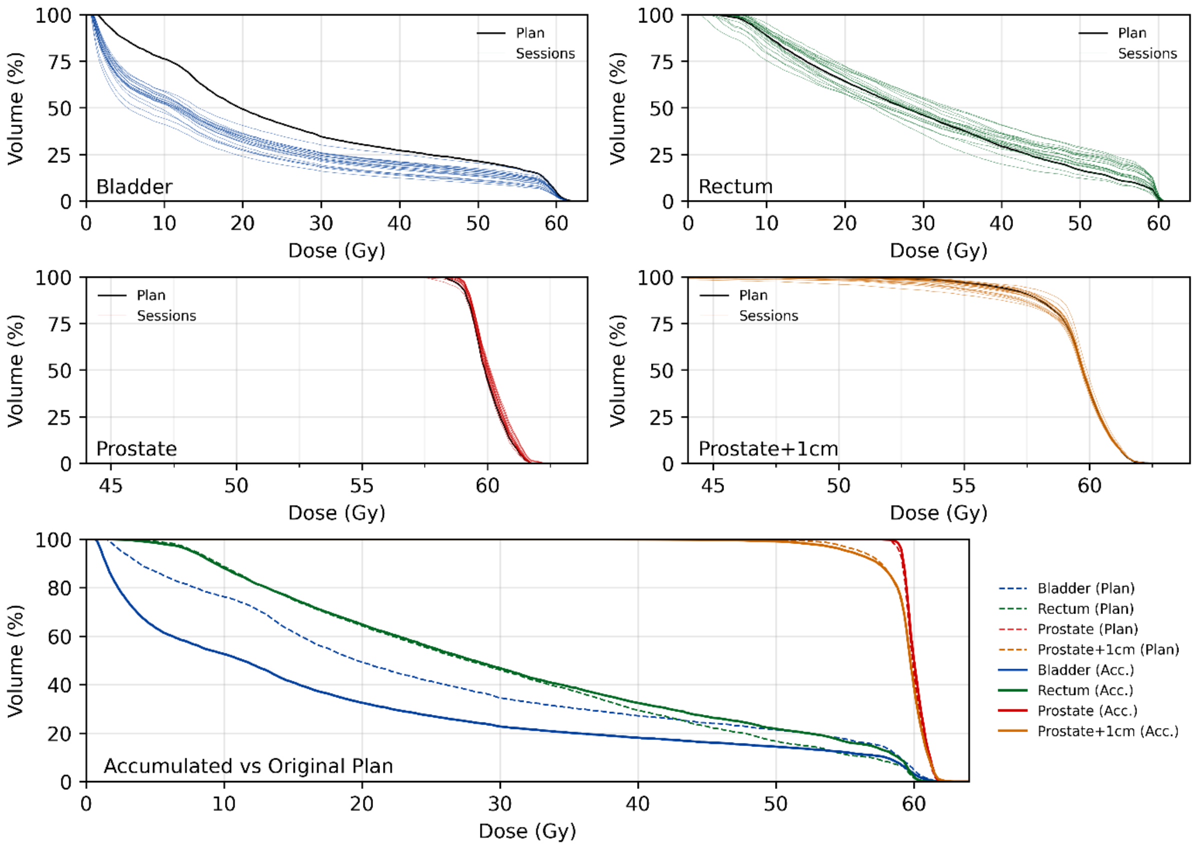

Figure S5. Accumulated DVH assessment for Patient P4.

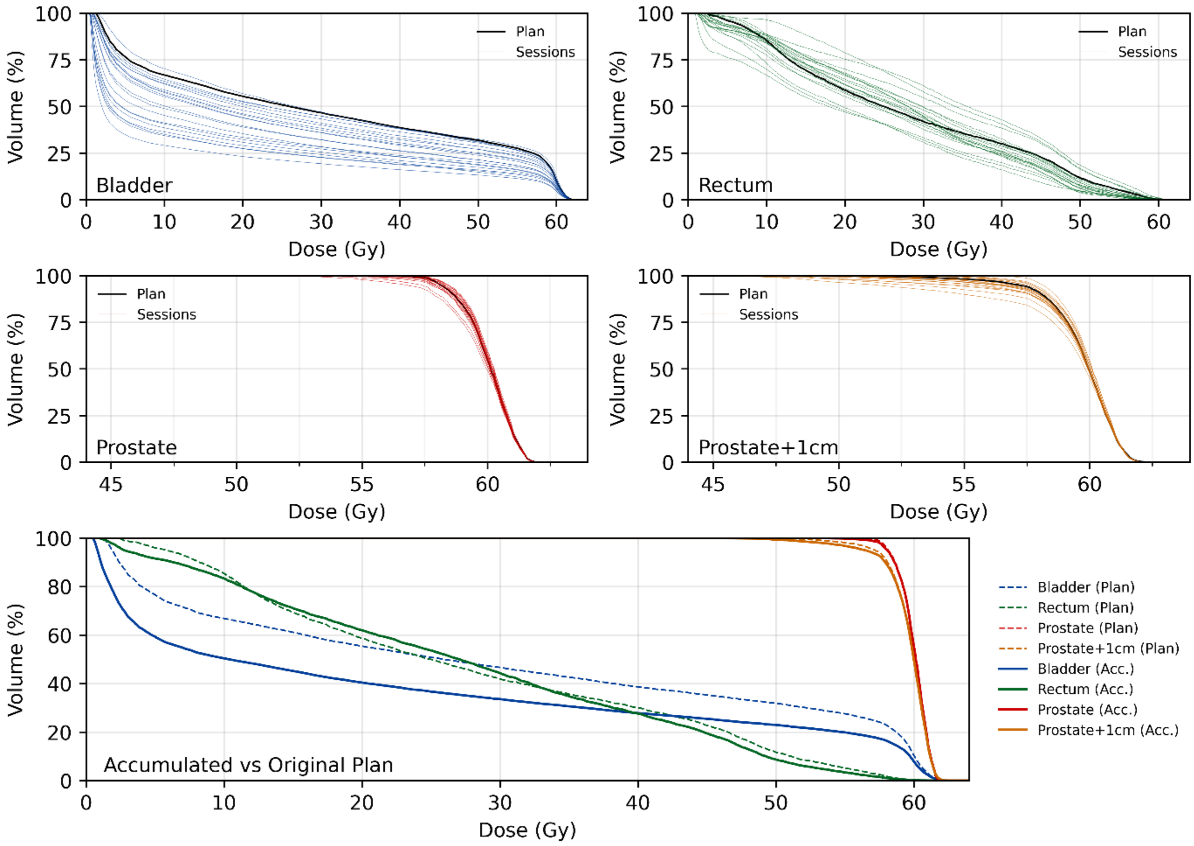

Figure S6. Accumulated DVH assessment for Patient P5.

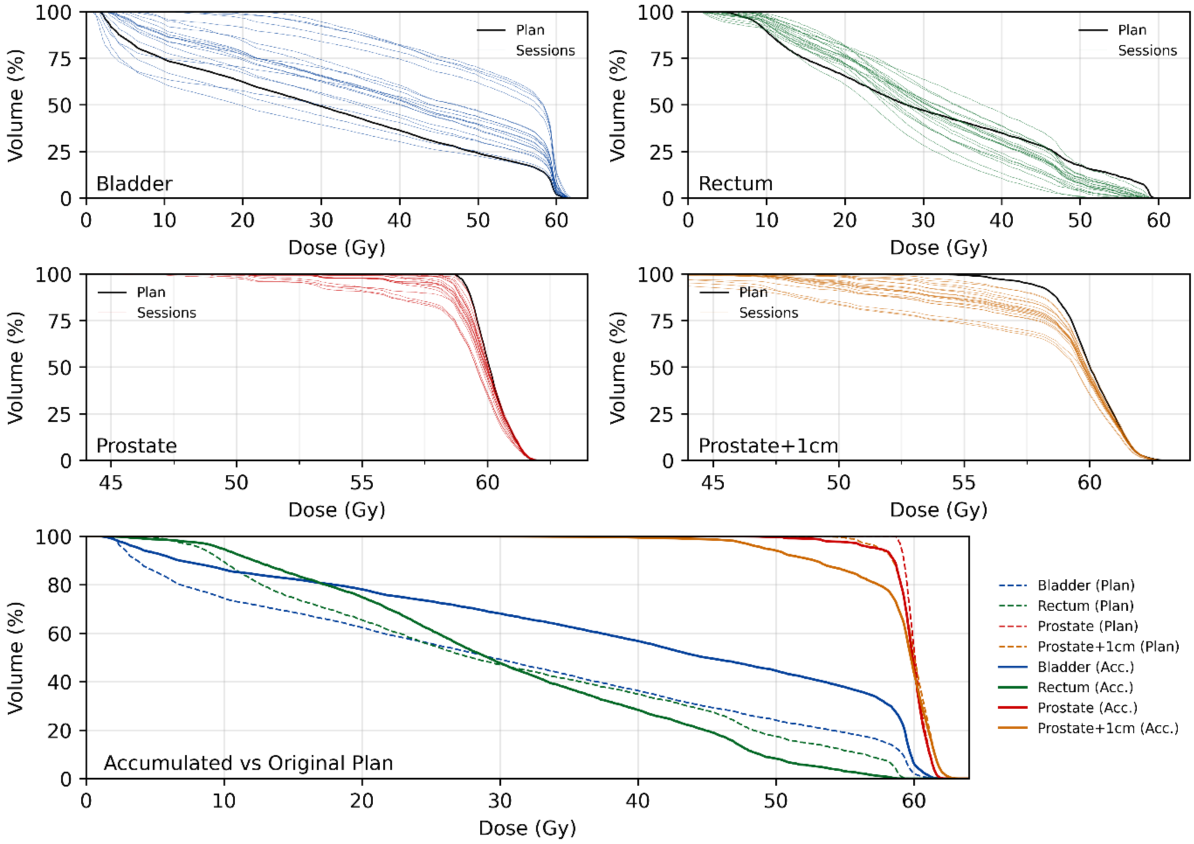

Figure S7. Accumulated DVH assessment for Patient P6.

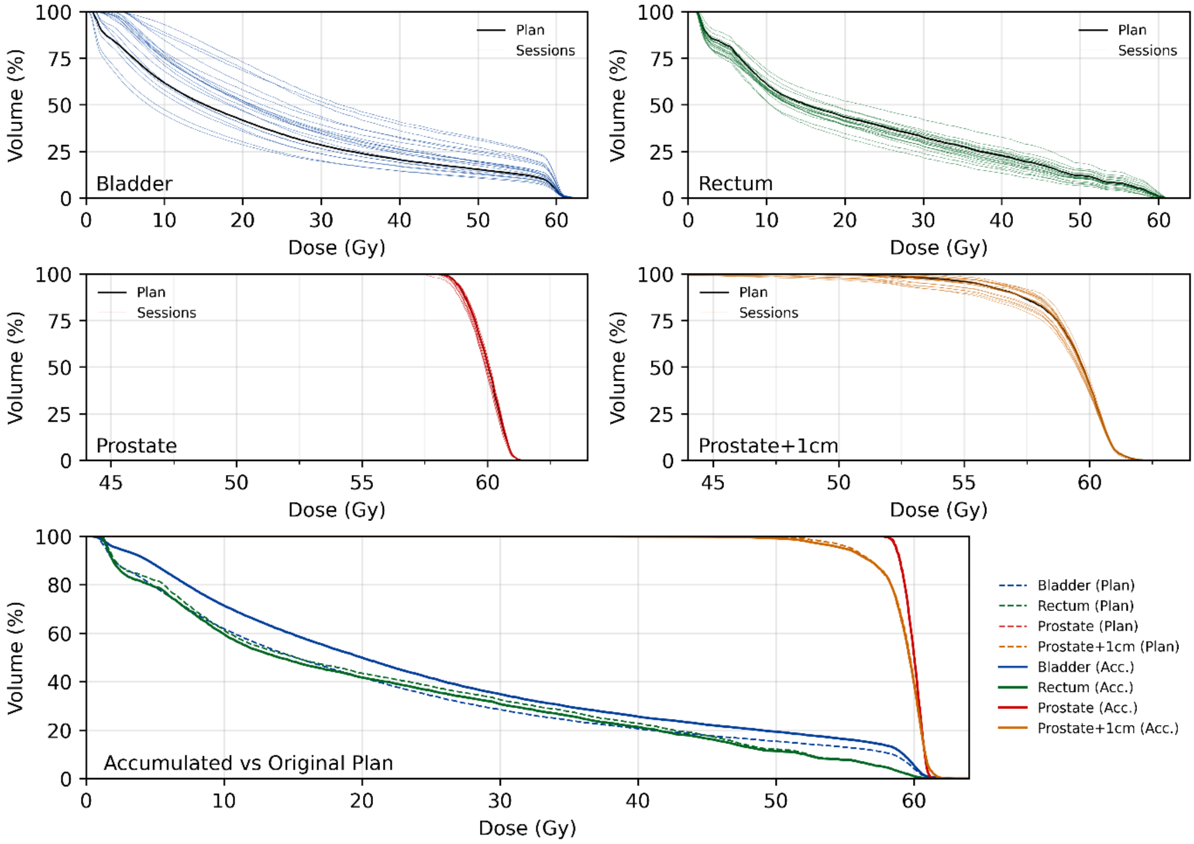

Figure S8. Accumulated DVH assessment for Patient P7.

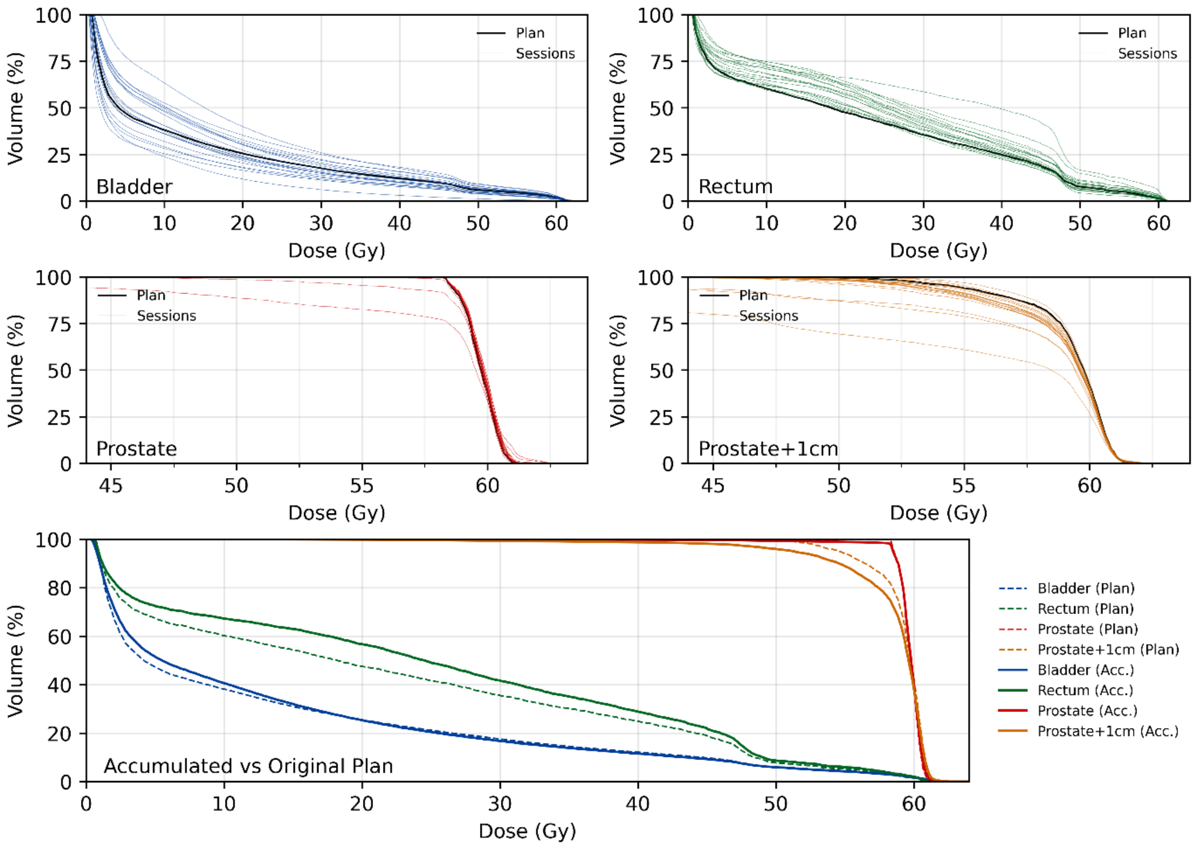

Figure S9. Accumulated DVH assessment for Patient P8.

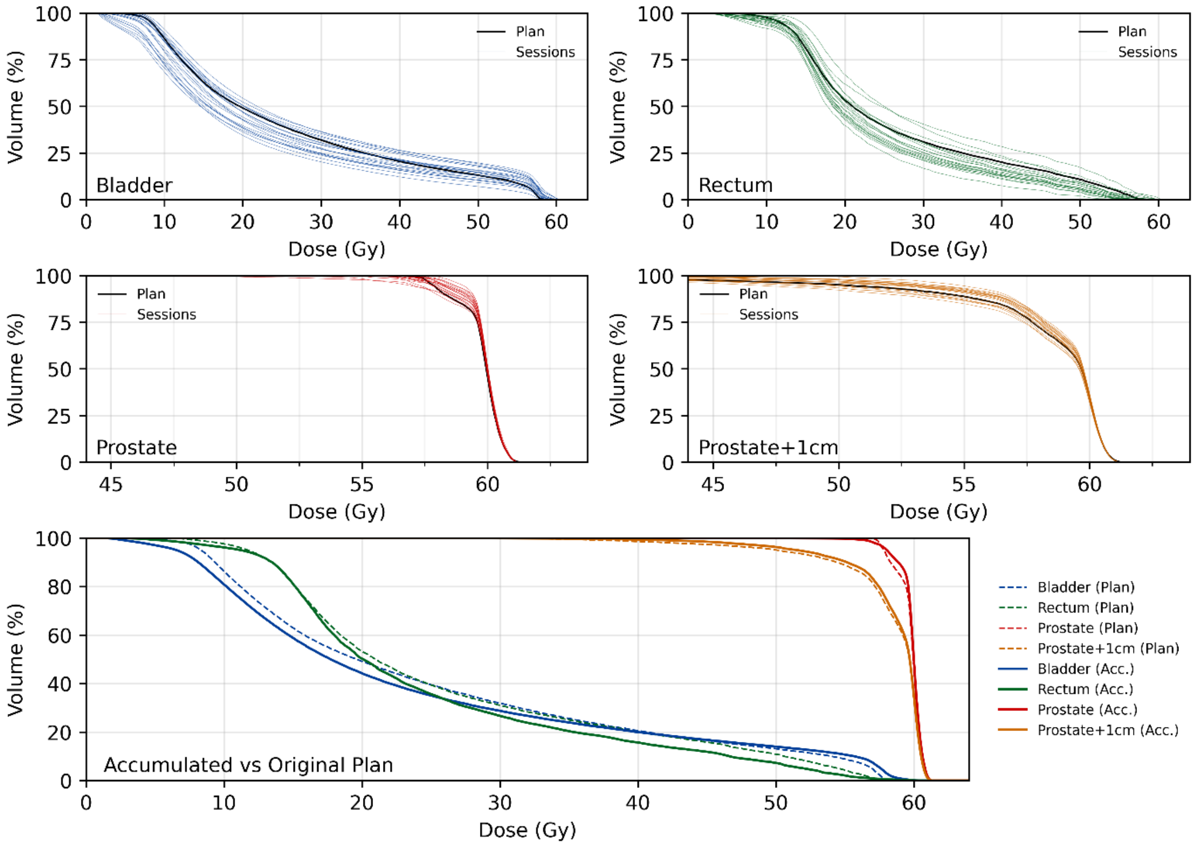

Figure S10. Accumulated DVH assessment for Patient P9.

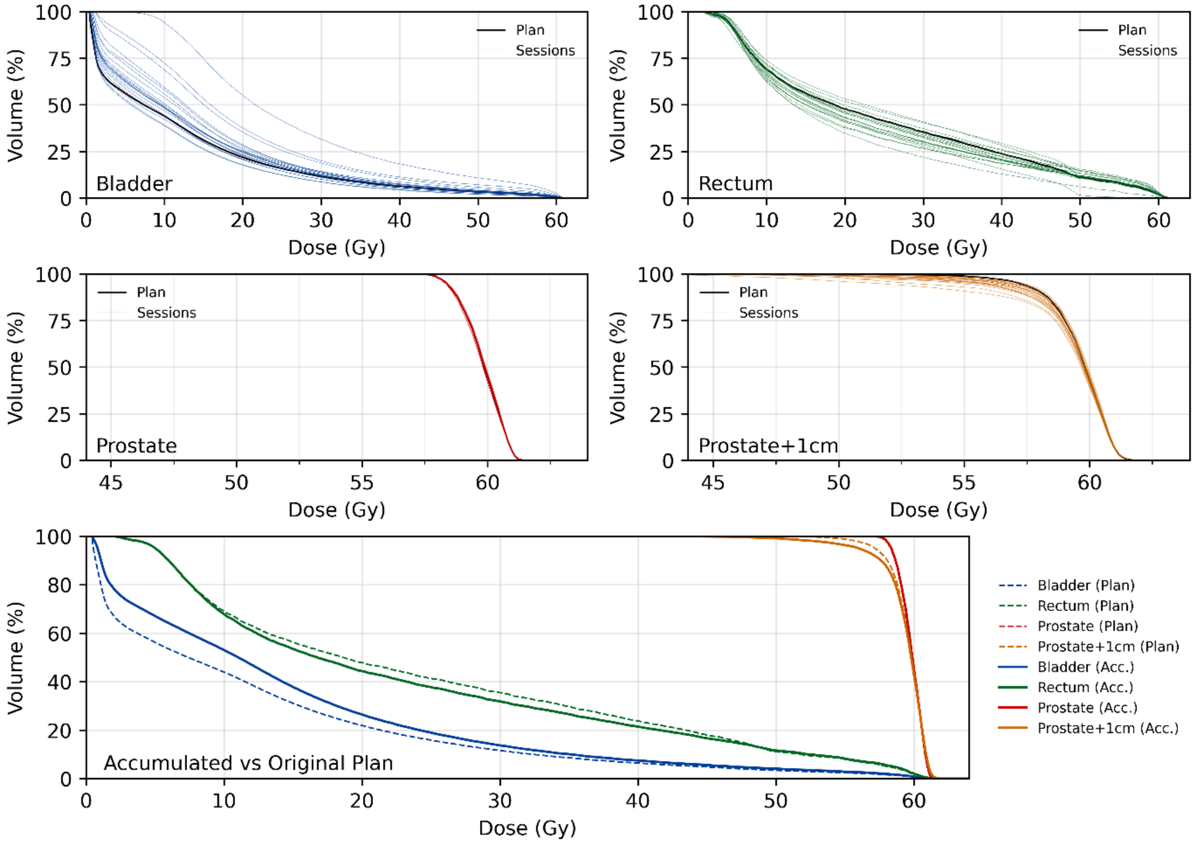

Figure S11. Accumulated DVH assessment for Patient P10.

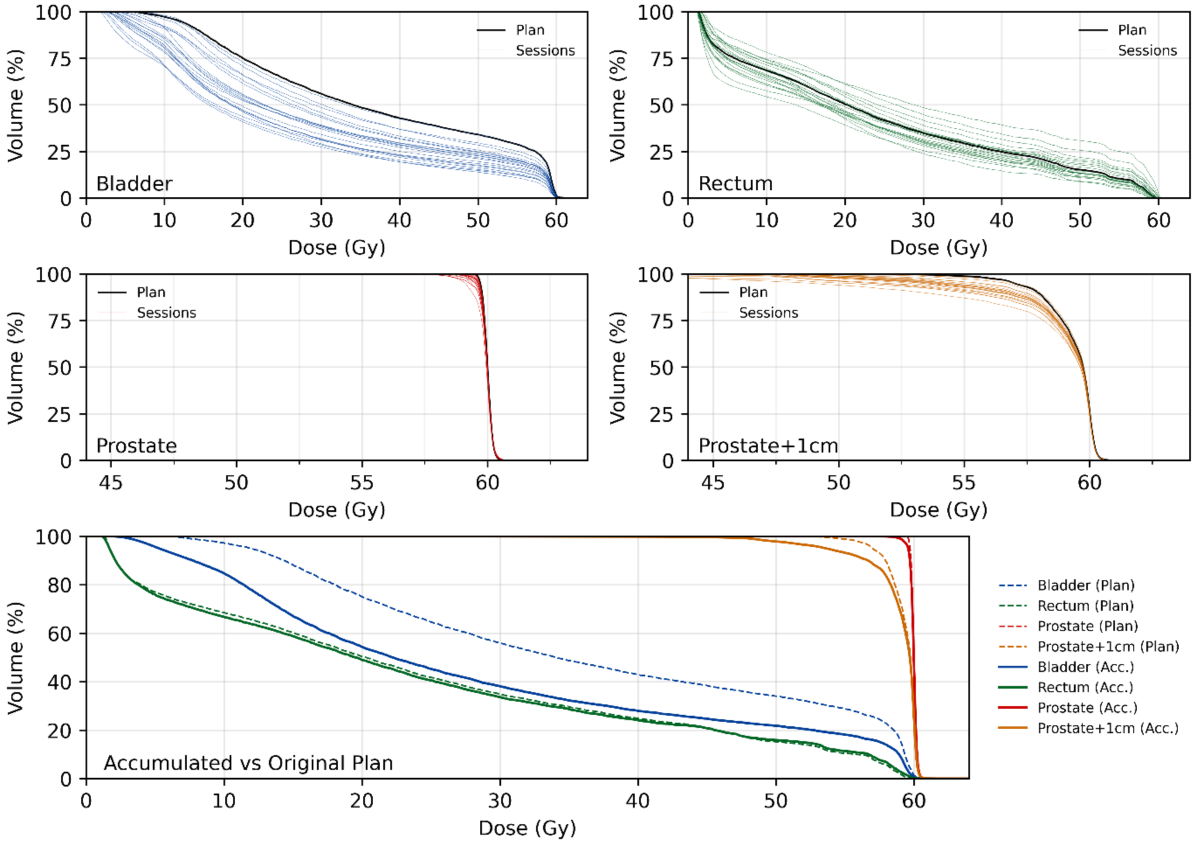

Figure S12. Accumulated DVH assessment for Patient P11.

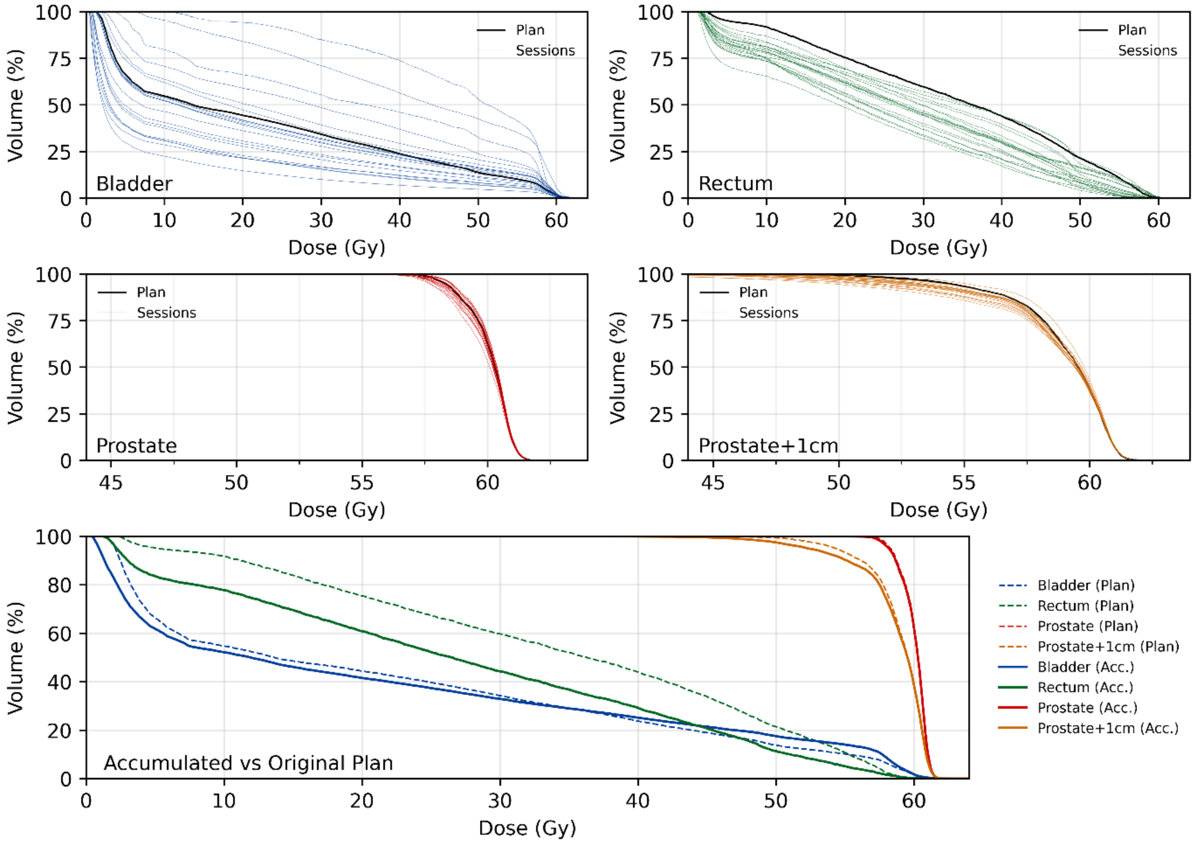

Figure S13. Accumulated DVH assessment for Patient P12.

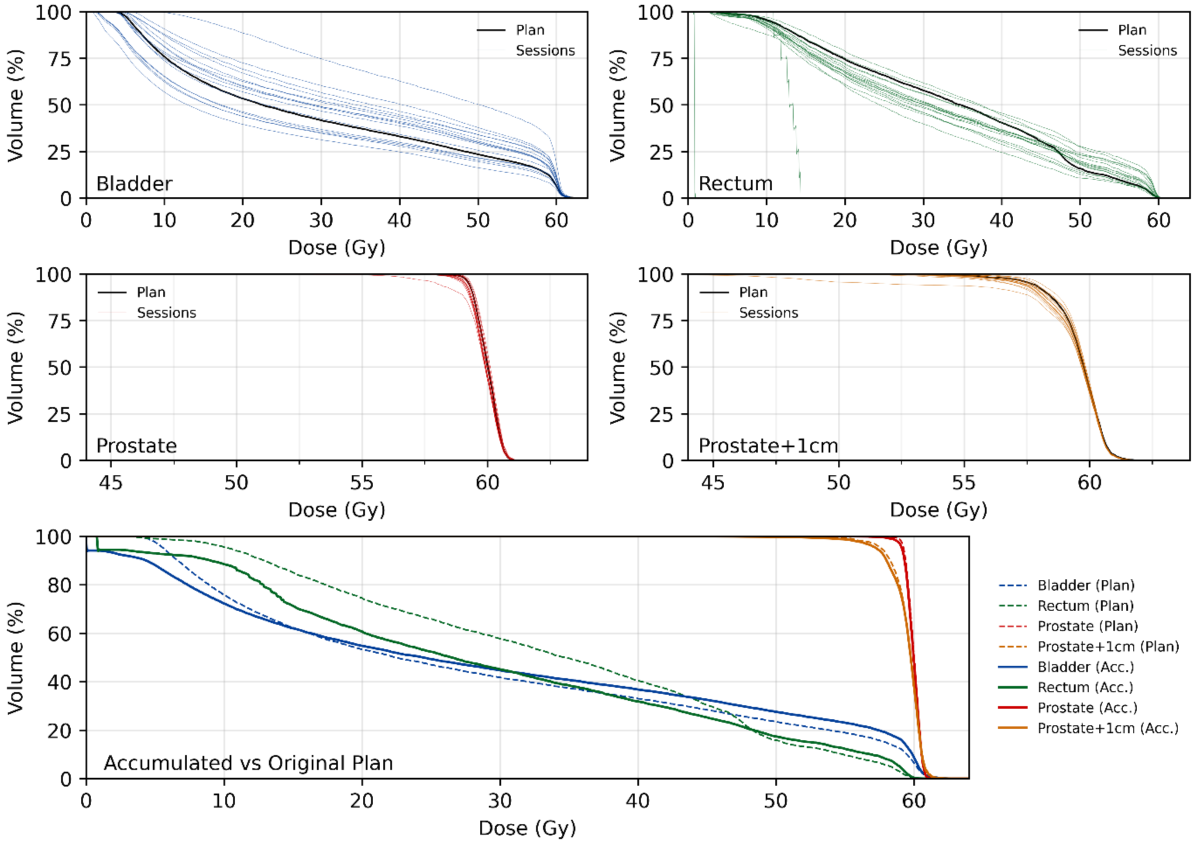

Figure S14. Accumulated DVH assessment for Patient P13.

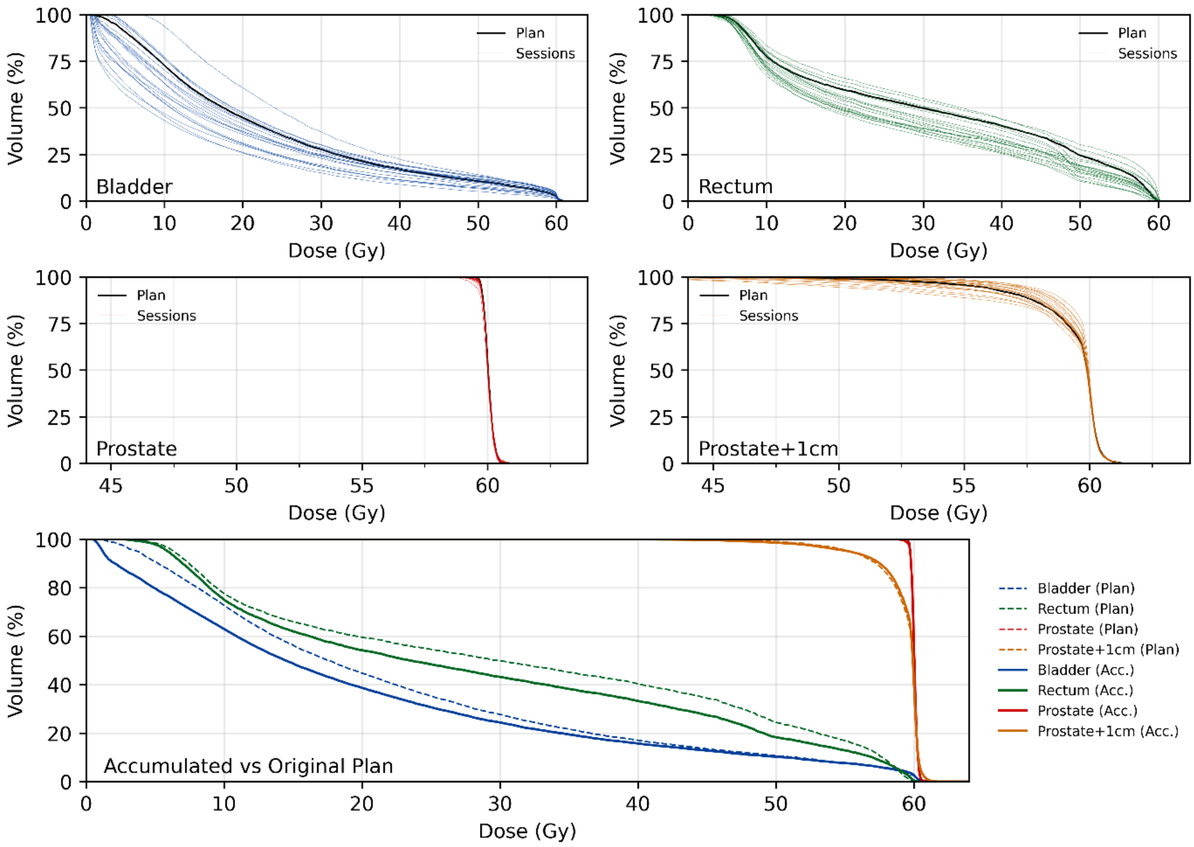

Figure S15. Accumulated DVH assessment for Patient P14.

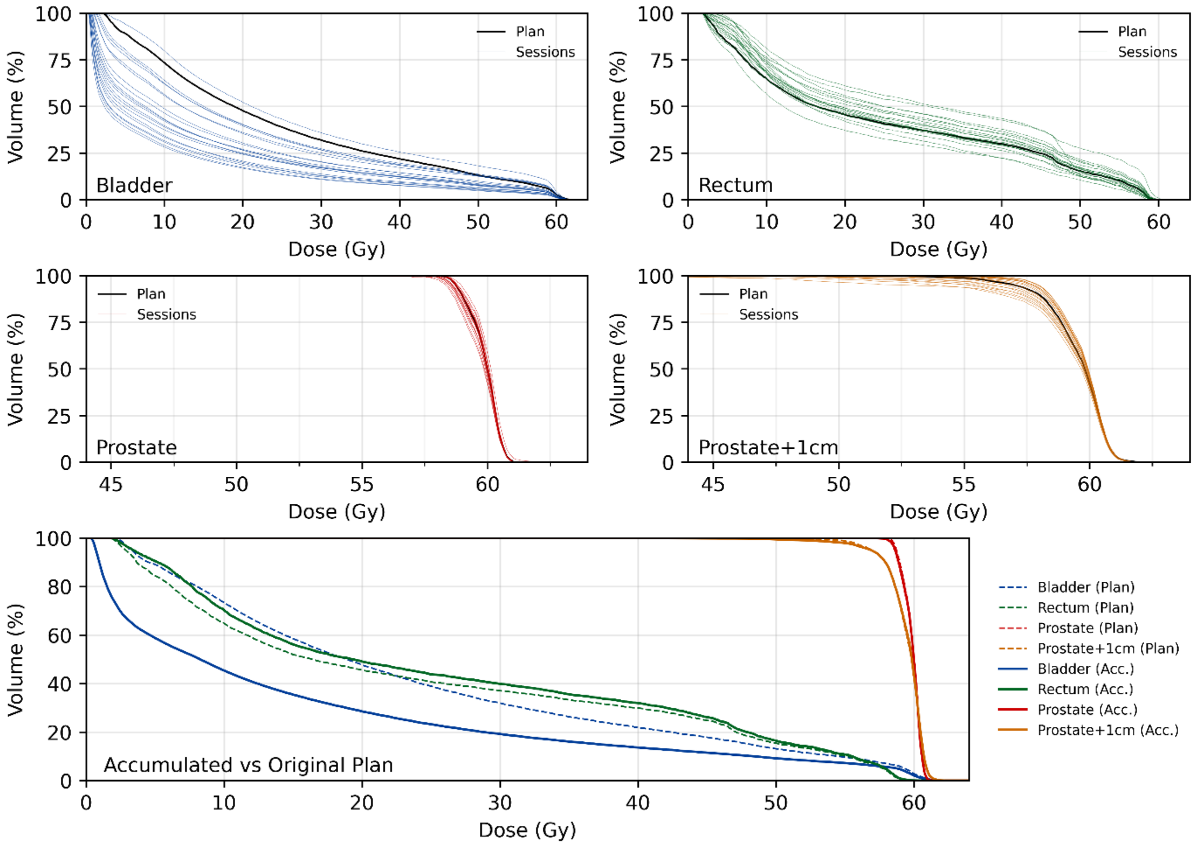

Figure S16. Accumulated DVH assessment for Patient P15.

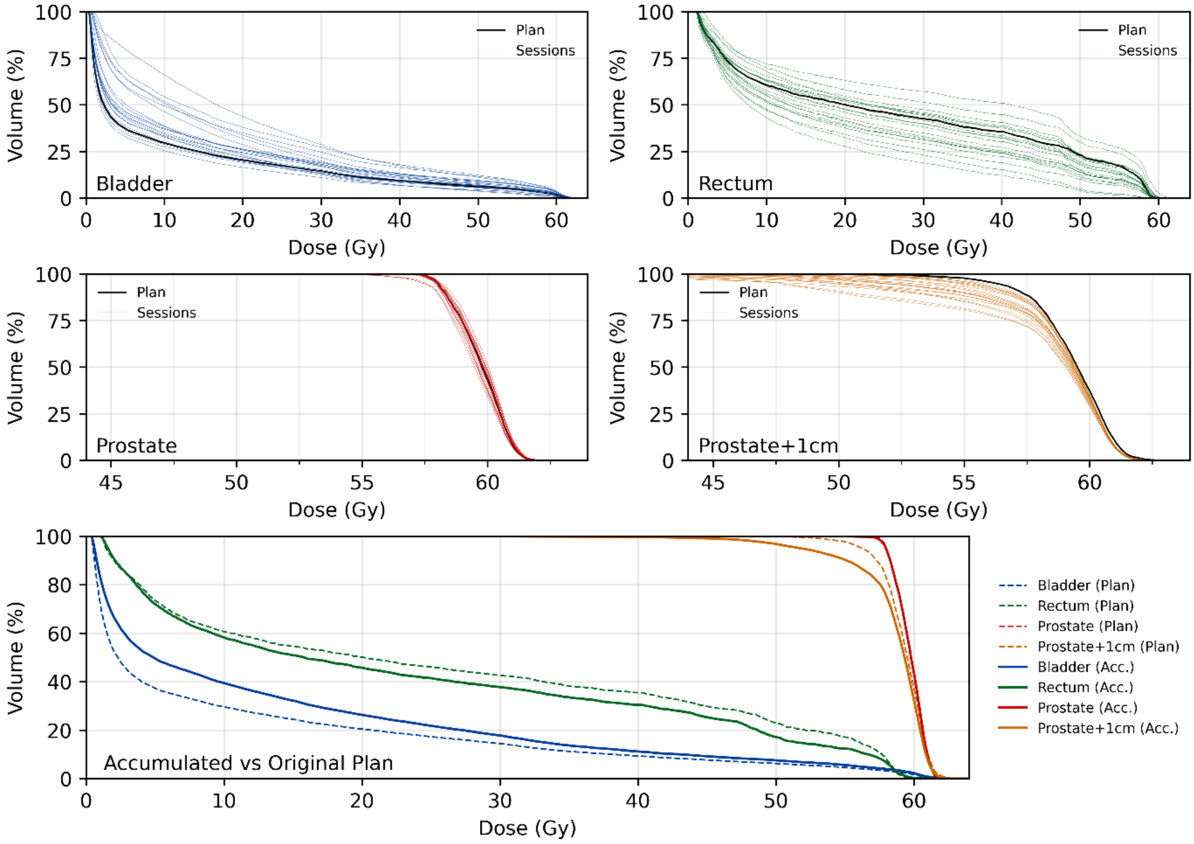

Figure S17. Accumulated DVH assessment for Patient P16.

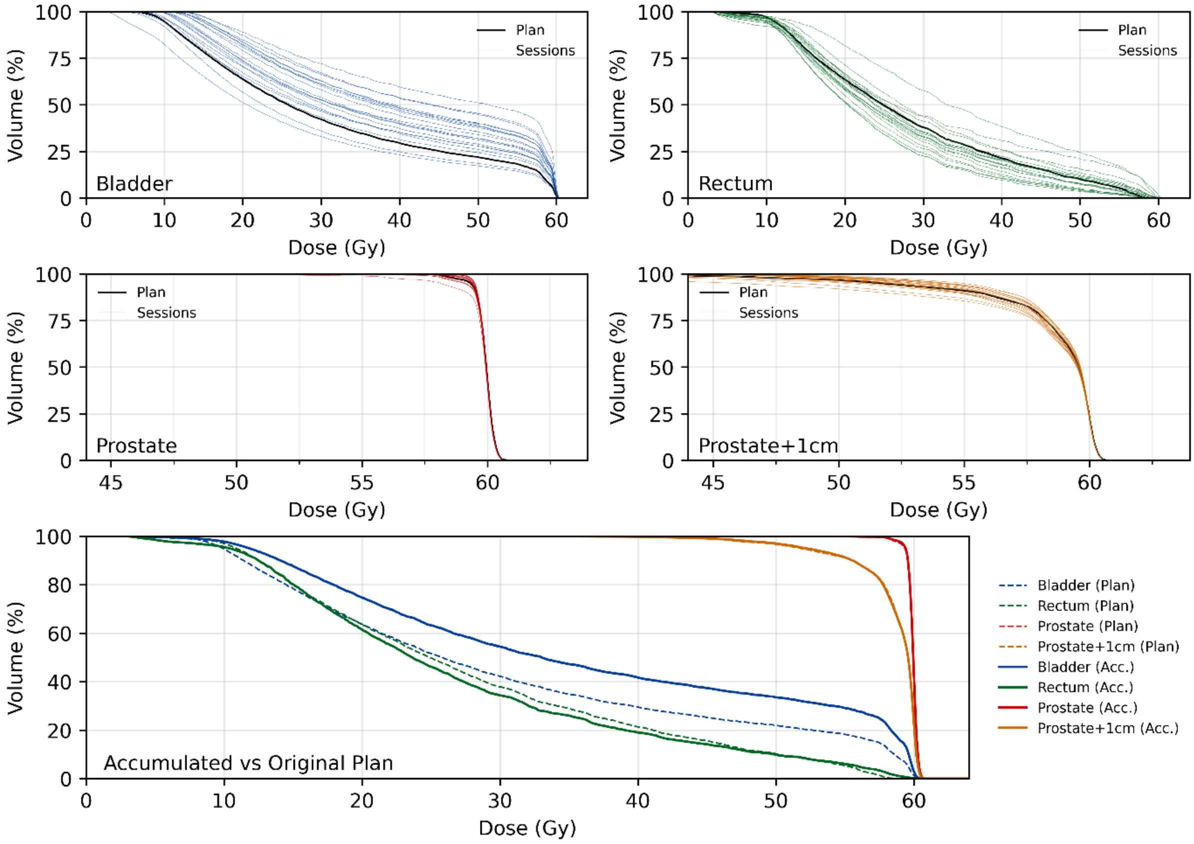

Figure S18. Accumulated DVH assessment for Patient P17.

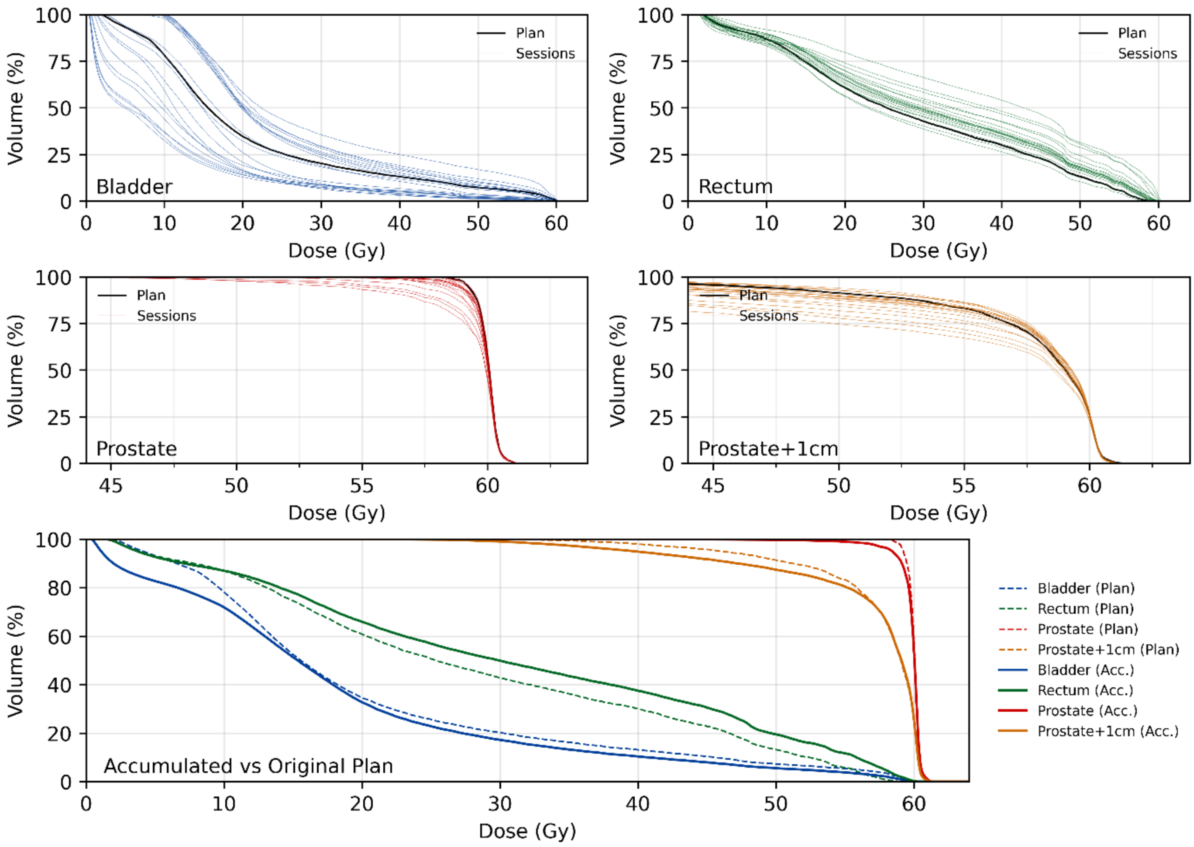

Figure S19. Accumulated DVH assessment for Patient P18.

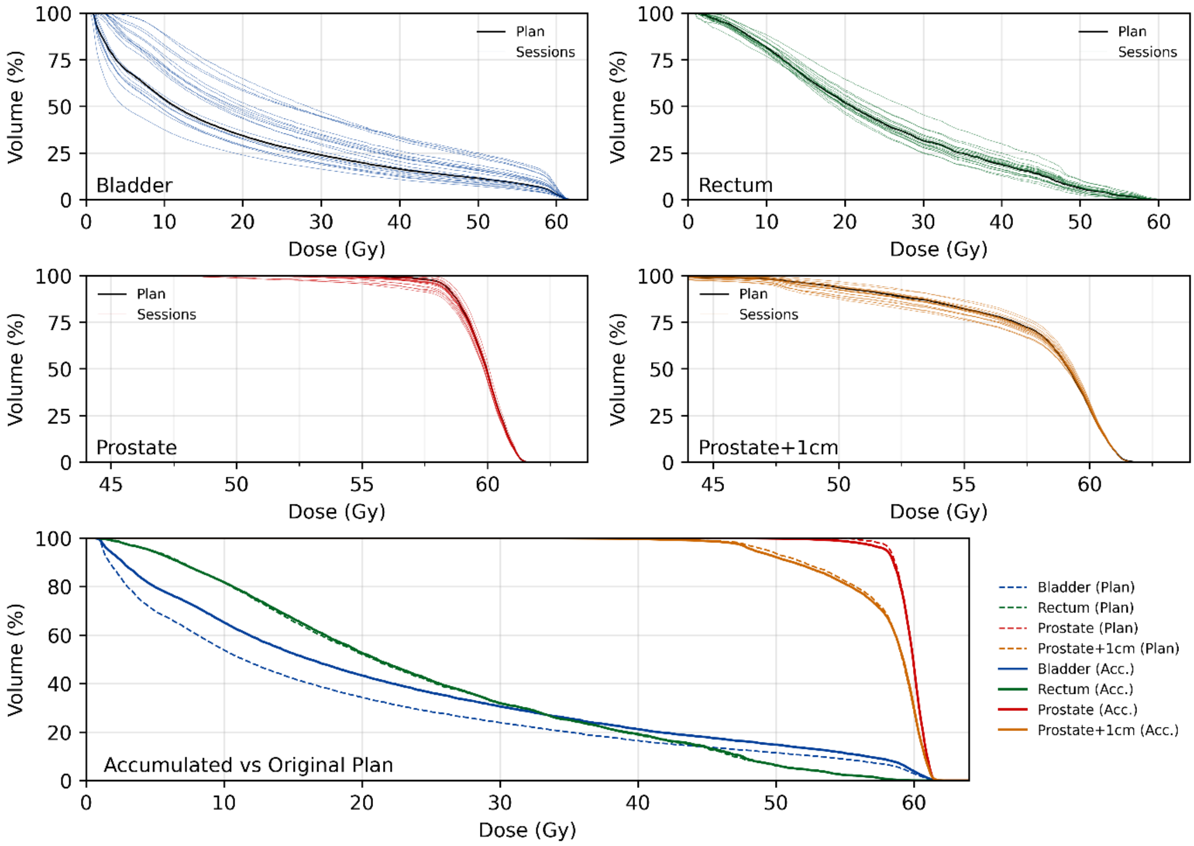

Figure S20. Accumulated DVH assessment for Patient P19.

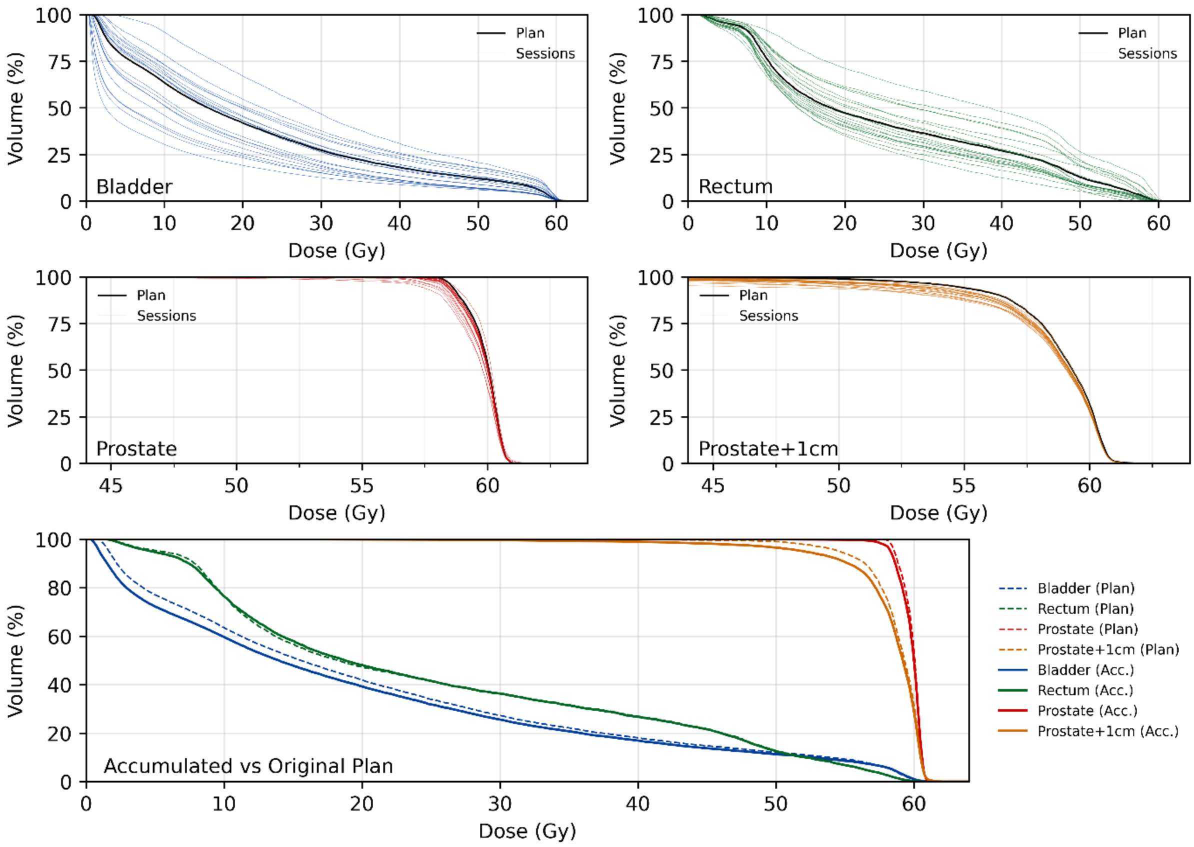

Figure S21. Accumulated DVH assessment for Patient P20.

Supplementary Material E – Validation of Dose Accumulation Against CBCT Recalculations

Table S1: Validation of our fast method with respect to results from CBCT-based dose recalculation. Results are presented for three representative cases, which were selected based on their body volume variation, quantified by the coefficient of variation (CV, Equation 2): P6 (minimal variation), P7 (second-highest variation), and P2 (maximal variation). Differences are presented for key dose–volume metrics of prostate and organs of interest. Negative values indicate lower estimates by our method compared to CBCT recalculation. pp denotes percentage points. The last three rows show the mean differences across all evaluated metrics for each structure type.

**Patient**	**Structure**	**Sessions**	**V_{20Gy}[%]-Difference in %**	**V_{50Gy}[%]-Difference in %**	**$D_{2\%}$[Gy]-Difference in %**	**$D_{50\%}$[Gy]-Difference in %**	**Mean[Gy]-Difference in %**
P6/ Low-CV	Prostate	20	-0.00± 0.0	-0.0 ± 0.0	-1.7 ± 0.5	-1.5 ± 0.4	-1.5 ± 0.4
P7/ High-CV	Prostate	20	0.0 ± 0.0	-0.01 ± 0.08	-0.5 ± 0.2	-0.3 ± 0.2	-0.4 ± 0.2
P2/ Max-CV (TEP)	Prostate	20	-0.0 ± 0.0	-0.00 ± 0.00	-0.4 ± 0.2	-0.2 ± 0.2	-0.1 ± 0.2
P6/ Low-CV	Bladder	20	-0.9 ± 0.7	-0.84 ± 0.69	-1.8 ± 0.6	-2.2 ± 1.9	-2.1 ± 1.2
P7/ High-CV	Bladder	20	0.3 ± 0.6	-0.73 ± 0.55	-1.3 ± 0.7	-0.3 ± 1.1	-0.2 ± 0.4
P2/ Max-CV (TEP)	Bladder	20	0.3 ± 0.2	0.17 ± 0.19	0.0 ± 0.4	1.1 ± 0.8	0.8 ± 0.5
P6/ Low-CV	Rectum	20	-0.8 ± 0.8	-1.05 ± 1.04	-1.2 ± 0.7	-2.5 ± 2.5	-1.9 ± 1.6
P7/ High-CV	Rectum	20	-0.5 ± 0.3	-0.42 ± 0.39	-0.6 ± 0.3	-1.1 ± 0.5	-0.8 ± 0.4
P2/ Max-CV (TEP)	Rectum	20	-0.1 ± 0.3	-0.09 ± 0.12	-0.5 ± 0.2	0.1 ± 1.3	0.0 ± 0.5
All (P6, P7 and P2)	Prostate	60	0.0 ± 0.0	-0.00 ± 0.05	-0.9 ± 0.7	-0.7 ± 0.7	-0.7 ± 0.7
	Bladder	60	-0.1 ± 0.8	-0.46 ± 0.69	-1.0 ± 1.0	-0.5 ± 1.9	-0.5 ± 1.4
	Rectum	60	-0.5 ± 0.6	-0.52 ± 0.76	-0.8 ± 0.6	-1.2 ± 2.0	-0.9 ± 1.3

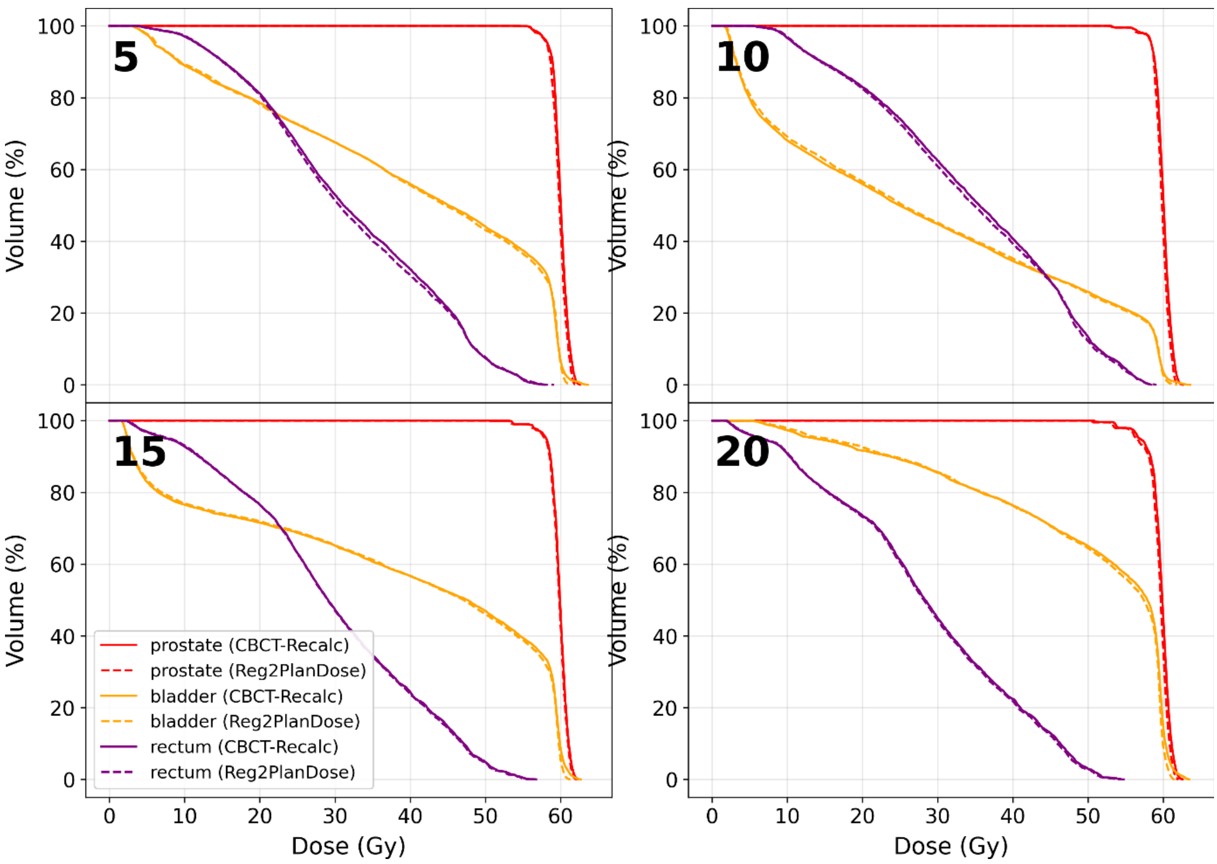

Figure S22. DVH comparison between CBCT-based dose recalculation (solid lines) and accumulated dose estimate (dashed lines) for Patient P6. Four representative fractions (5, 10, 15, and 20) are shown. This patient exhibited minimal anatomical variation (CV = 0.62 %).

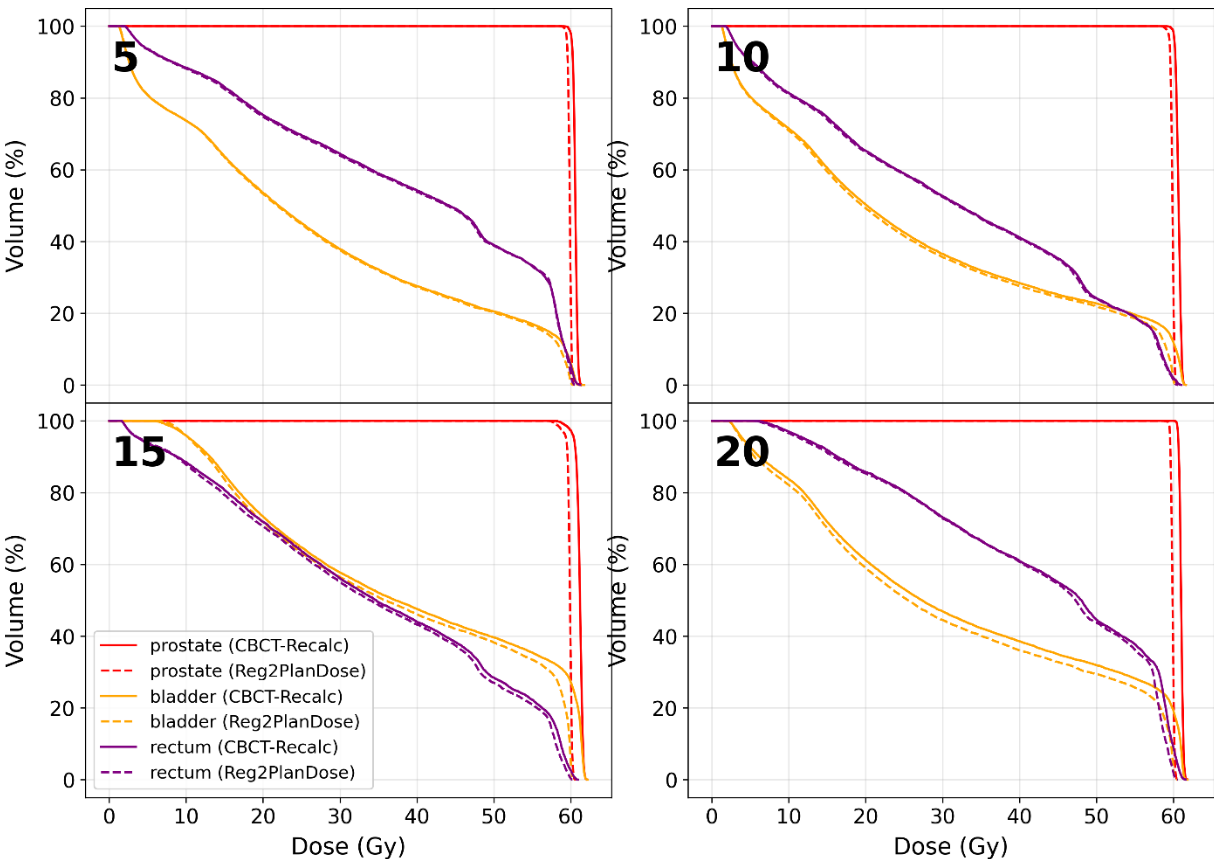

Figure S23. DVH comparison between CBCT-based dose recalculation (solid lines) and accumulated dose estimate (dashed lines) for Patient P7. Four representative fractions (5, 10, 15, and 20) are shown. This patient exhibited moderate anatomical variation (CV = 2.67 %).

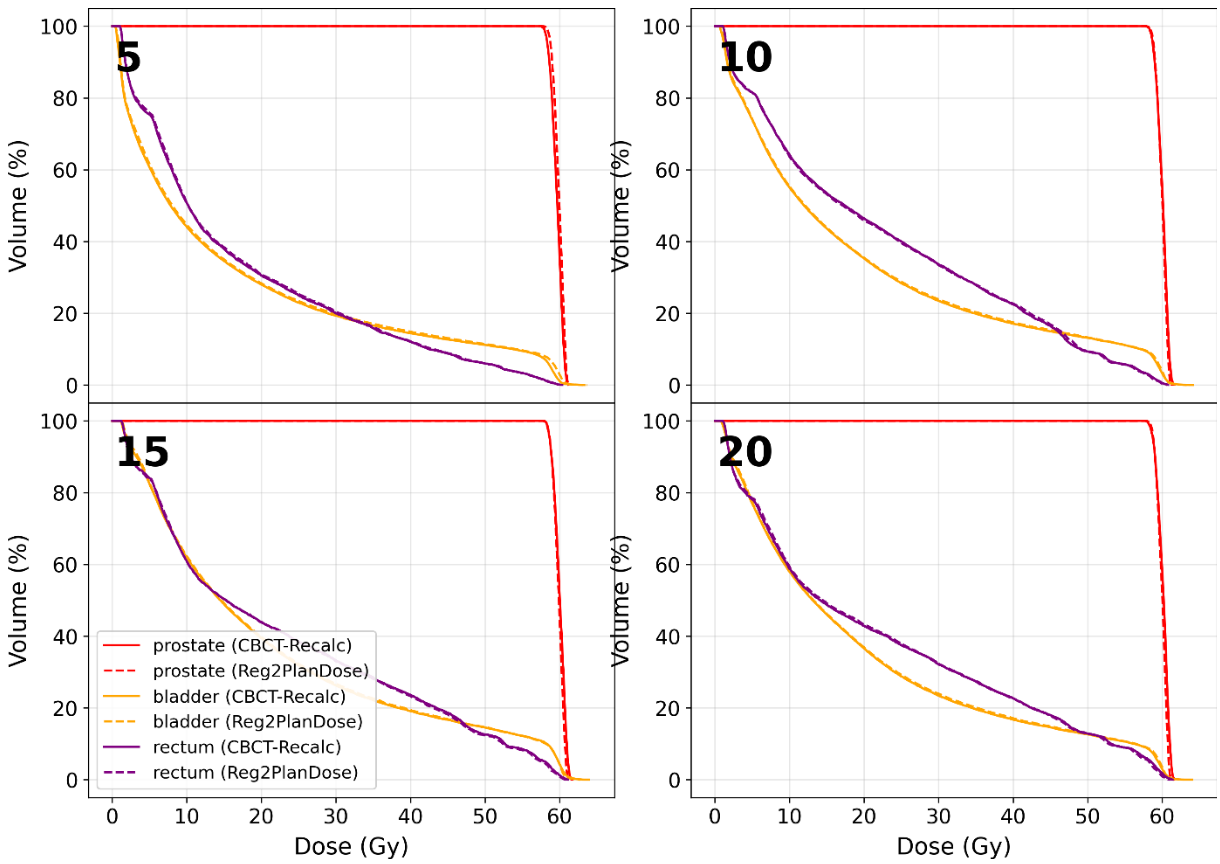

Figure S24. DVH comparison between CBCT-based dose recalculation (solid lines) and accumulated dose estimate (dashed lines) for Patient P2. Four representative fractions (5, 10, 15, and 20) are shown. This patient exhibited the largest anatomical variation (CV = 3.84 %). A right-sided hip prosthesis caused CBCT imaging artifacts.

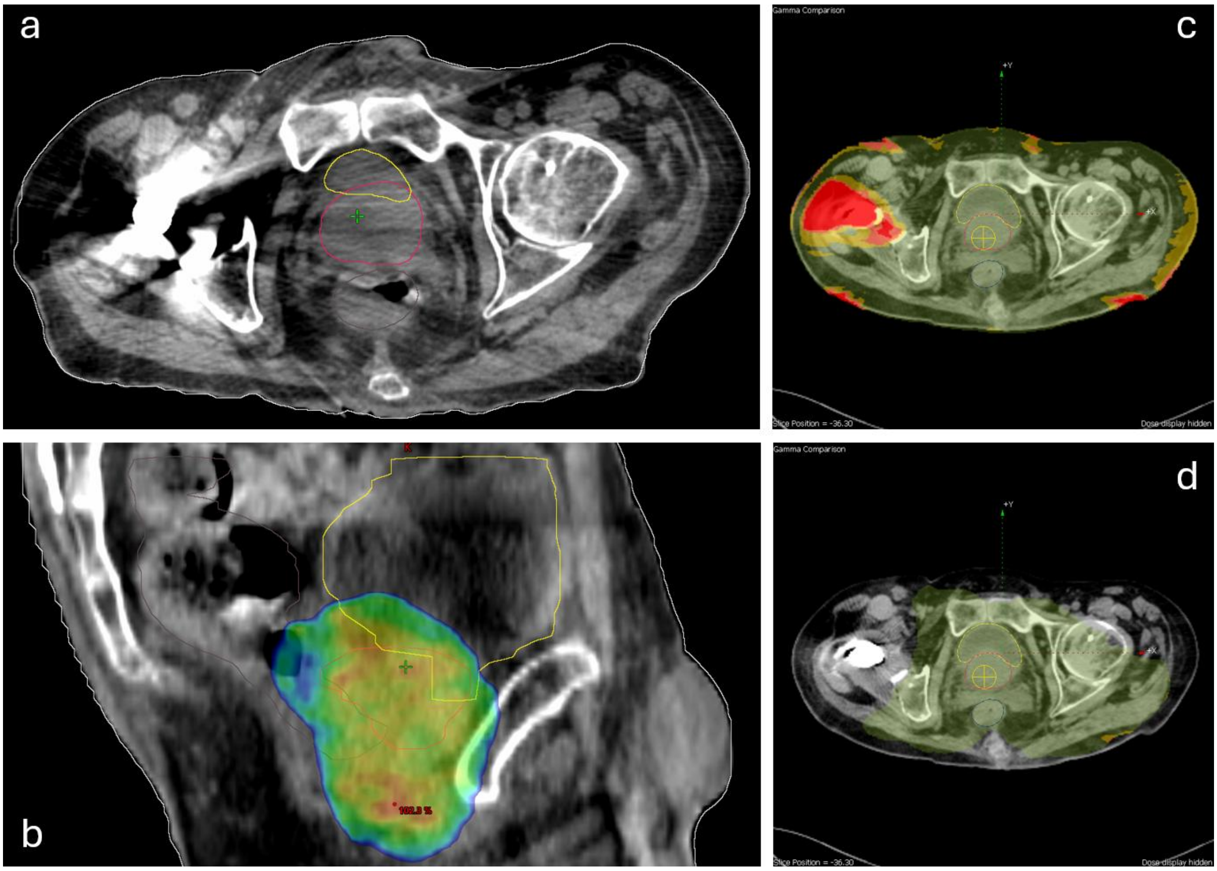

Figure S25: Illustration of the impact of a unilateral hip prosthesis (patient P2) on CBCT-based contouring and dose recalculation.

(a) Axial CBCT slice showing pronounced streaking artifacts caused by the right-sided hip prosthesis.

(b) Corresponding axial slice with CBCT-based dose recalculation displayed as colorwash (from 95 % of the prescription dose to maximum dose), demonstrating preserved target coverage despite visible artifacts.

(c) Gamma comparison between the planned dose distribution calculated on the planning CT (with metal artifact reduction) and the CBCT-based recalculated dose using 3 %/3 mm criteria with a 1 % dose threshold. Localized gamma failures are predominantly observed outside clinically relevant high-dose regions.

(d) Gamma comparison using identical criteria but applying a 30 % dose threshold, showing high overall agreement and indicating that remaining discrepancies are largely confined to low-dose regions and areas affected by artifacts.
